# Cell Development Deficiency and Gene Expression Dysregulation of Trisomy 21 Retina Revealed by Single-Nucleus RNA Sequencing

**DOI:** 10.3389/fbioe.2020.564057

**Published:** 2020-09-23

**Authors:** Fang-Yuan Hu, Feng-Juan Gao, Ping Xu, Sheng-Hai Zhang, Ji-Hong Wu

**Affiliations:** ^1^Eye Institute and Department of Ophthalmology, Eye & ENT Hospital, Fudan University, Shanghai, China; ^2^NHC Key Laboratory of Myopia (Fudan University); Key Laboratory of Myopia, Chinese Academy of Medical Sciences, Shanghai, China; ^3^Shanghai Key Laboratory of Visual Impairment and Restoration, Shanghai, China

**Keywords:** trisomy 21 retina 1, single nucleus RNA sequencing 2, cellular compositions 3, differentiation process 4, communication networks 5

## Abstract

Retina is a crucial tissue for capturing and processing light stimulus. It is critical to describe the characteristics of retina at the single-cell level for understanding its biological functions. A variety of abnormalities in terms of morphology and function are present in the trisomy 21 (T21) retina. To evaluate the consequences of chromosome aneuploidy on retina development, we identified the single-cell transcriptional profiles of a T21 fetus and performed comprehensive bioinformatic analyses. Our data revealed the diversity and heterogeneity of cellular compositions in T21 retina, as well as the abnormal constitution of T21 retina compared to disomic retina. In total, we identified seven major cell types and several subtypes within each cell type, followed by the detection of corresponding molecular markers, including previously reported ones and a series of novel markers. Through the analysis of the retinal differentiation process, subtypes of retinal progenitor cells (RPCs) exhibiting the potential of different retinal cell-type commitments and certain Müller glial cells (MGs) with differentiating potency were identified. Moreover, the extensive communication networks between cellular types were confirmed, among which a few ligand–receptor interactions were related to the formation and function of retina and immunoregulatory interactions. Taken together, our data provides the first ever single-cell transcriptome profiles for human T21 retina, which facilitates the understanding on the dosage effects of chromosome 21 on the development of retina.

## Introduction

Retina is a highly specialized neural tissue that contains a variety of neurocyte types, which can sense light and initiate image processing. Retinal development begins with the eyeball establishment mediated by SHH and SIX3 proteins, followed by the development of optic nerve vesicles regulated by PAX6 and LHX2 proteins (Heavner and Pevny, 2012). The vesicles produce three structures, namely, the neural retina, the retinal pigment epithelium (RPE), and the rod. The neural retina contains retinal progenitor cells (RPCs), which produce seven cell types of the retina (Masland, 2012). It has been reported that the development of retina can be divided into three periods according to RNA sequencing (RNA-Seq). First of all, in the early retina, progenitor cell proliferation and ganglion cell production are predominant (Rheaume et al., 2018). The second period is characterized by the appearance of horizontal cells (HCs) and amacrine cells (ACs), during which the expression of synapse-related genes is significantly upregulated. The third period exhibits the production and differentiation of photoreceptors (PRs), bipolar cells (BCs), and Müller glial cells (MGs) (Baker et al., 2014; Cepko, 2015; Hoshino et al., 2017).

Trisomy 21 (T21), also known as Down’s syndrome, is the most common disease caused by abnormal chromosome dosage, with a worldwide incidence reaching 1 in 700 (Stoll et al., 1998). Patients with Down’s syndrome are clinically manifested by congenital heart defects, premature aging, early onset of Alzheimer’s disease, leukemia, and retinal developmental abnormality (Malt et al., 2013). One of the common clinical complications in children with Down’s syndrome is ophthalmic disease. It has also been reported that the abnormalities in neurological pathway are found in T21 patients (Laguna et al., 2013). In addition, adults with Down’s syndrome have significant visual impairment relative to the disomic individuals (Krinsky-McHale et al., 2014).

Although it is known that T21 individuals suffer from retina diseases, the cellular compositions and related molecular regulatory mechanisms in the T21 retina remain largely unknown. The single-cell RNA-sequencing (scRNA-seq) technique has been widely employed to the study of retinal transcriptome in human, mouse, monkey, and chicken (Macosko et al., 2015; Shekhar et al., 2016; Laboissonniere et al., 2017; Lu et al., 2020), which has comprehensively described the transcription profiles of retina cells under disomic condition and identified the gene regulatory networks of neurogenesis and cell fate specification (Gawad et al., 2016). To explore the heterogeneity of T21 retina, we performed single-nucleus RNA sequencing (snRNA-seq) analysis on the retina from a fetus with a gestational age of 22 weeks (GW22). According to the study of human retinal development in the mid-gestational stages (GW14 to GW27), the cell types of retina are sequentially specified by RPCs based on the analysis of time-series scRNA-seq data. Particularly, GW22 is the start point of PR emergence (Lu et al., 2020). In this study, we examined the gene expression profiles of T21 retinal cells and identified major retina cell types, further exploring the significant influence of the redundancy of chromosome 21 in certain cell types.

## Materials and Methods

### Sample and Ethics Statement

T21 human fetal retina collection and research were approved by the Medical Ethics Committee of Shiyan Taihe Hospital (201813). The gestation was selectively induced to end. The informed consent forms were signed under the ISSCR guidelines for fetal tissue donation and in strict observance of the legal and institutional ethical regulations on the elective pregnancy termination of patients, after her decision to legally terminate the pregnancy but before the abortive procedure. All tissue samples used in this study were not previously involved in any other procedures. All protocols were in compliance with the “Interim Measures for the Administration of Human Genetic Resources” administered by the Chinese Ministry of Health.

Trisomy of chromosome 21 of the male fetus in this study was diagnosed by specialists in Shiyan Taihe Hospital based on the karyotyping with amniocentesis. Under the guidance of B-ultrasound, the needle was inserted into the amniotic fluid through the abdomen of pregnant women, and the amniotic fluid was extracted for chromosome analysis of fetal cells. The fetus had a karyotype of 47,XY, + 21.

### Tissue Sample Collection and Dissociation

We collected a retina from the left eye of a T21 human fetus (22 weeks’ gestation). Gestational age was measured in weeks from the first day of woman’s last menstrual cycle to the sample collecting date. The T21 human fetal retina sample was collected into the Stroke-physiological saline solution. A rapid hemi-section was performed to remove the vitreous and the anterior. The retina was carefully dissected free from the posterior eyecup and then flash frozen in liquid nitrogen. The tissue was provided with de-identified medical records including time and cause of death. The time between death and tissue collection was 3 h.

### Single-Cell cDNA Library Preparation and High-Throughput Sequencing

Flash-frozen tissue was homogenized in 2 mL ice-cold lysis buffer (10 mM Tris–HCl, pH 7.4/10 mM NaCl, 3 mM MgCl_2_, 0.1% NP40, protease inhibitors) and then dounced in an RNase-free 2-ml glass dounce (D8938-1SET, Sigma-Aldrich, America) 15 × with a loose pestle and 15 × with a tight pestle on ice. Homogenization was transferred through a 40-μm filter (352340 BD, Becton, Dickinson and Company, America) to remove the block mass. The cell filtrate was proceeded for density gradient centrifugation. Then, a 400-μL cell filtrate was mixed with 400 μL 50% iodixanol solution in 2 mL lo-Bind tubes (Z666556-250EA, Sigma-Aldrich, America). 29% iodixanol solution and 35% iodixanol solution were layered to the bottom of tube and centrifuged at 3,000 *g*, 4°C for 30 min.

Nuclei were resuspended in ice-cold 1 × PBS (10010-031 GIBCO, Thermo Fisher Scientific, Shanghai China) containing 0.04% BSA and spun down at 500 g for 5 min. The supernatant was discarded, and the regular-bore pipette tip was used to gently pipette 50 μL ice-cold 1 × PBS, containing 0.04% BSA and 0.2 U/μl RNase inhibitor, into the cell pellet. The nucleus concentration was determined using a hemocytometer (101010, Shanghai Qiujing Biochemical Reagent Instrument Company, Shanghai China) and then loaded on a Chromium Single Cell Controller (10x Genomics, Shanghai China) to generate single-cell Gel Bead-In-EMulsions (10 × Genomics, Shanghai China) using the Single Cell 3′ Library and Gel Bead Kit V2 (120237, 10x Genomics, Shanghai China). Captured cells released RNA and were barcoded in individual GEMs. Following the manufacturer’s instructions (120237, 10x Genomics), a library was generated from the donor sample. The indexed library was converted by the MGIEasy Lib Trans Kit (1000004155, MGI, China) and sequenced on the MGISEQ 2000 (MGI, China) platform with paired-end 26bp + 100bp + 8bp (PE26 + 100 + 8).

### Preprocessing and Quality Control of snRNA-Seq Data

We first used Cell Ranger 2.0.0 (10X Genomics, Shanghai, China) to process raw sequencing data and then applied Seurat (Satija et al., 2015)^1^ for downstream analysis. Before we started the downstream analysis, we focused on four filtering metrics to guarantee the reliability of our data (Supplementary Figures 1B–D). (1) We filtered out genes that were detected in less than 0.1% of total cell number to guarantee the reliability of each gene. (2) We filtered out cells whose percentage of expressed mitochondrial genes was greater than 10%. (3) We filtered out cells whose Unique Molecular Identifier (UMI) counts were either less than or greater than one IQR distance outer of the quartiles of UMI counts. Finally, we used the house keeping genes from Protein Alta^2^ to verify the reliability of our data.

### Analysis of Heterogeneity in Each Tissue and Cell Line

The heterogeneity of the retina sample was determined using Seurat R package (Satija et al., 2015) (see text footnote 1). Then, the significant principal component (PCs) was determined using the JackStrawPlot function. The top twelve PCs were used for cluster identification with resolution 1.0 using the k-nearest neighbor (KNN) algorithm and visualization using the t-Distributed Stochastic Neighbor Embedding (t-SNE) algorithm. Cell types were assigned by the expression of known cell-type markers (Supplementary Table 1) and functional enrichment analyses (Supplementary Table 2). The FindAllMarkers function in Seurat was used to identify marker genes for each cluster using default parameters. To remove the cell-cycle effects in clustering and cell-cycle analyses, we collected 42 genes and 53 genes related to the S phase and G2/M phase, respectively (Supplementary Table 3) (Kowalczyk et al., 2015; Tirosh et al., 2016). For clustering, each cell was assigned a score to describe its cell-cycle state by the CellCycleScoring function in Seurat according to the expression of these genes (Supplementary Figure 1A). Subsequently, the cell-cycle effect was regressed out based on the scores, leading to a more accurate clustering result. For cell-cycle analysis, cells were determined to be quiescent (G1 stage) if their S score < 0 and G2/M score < 0; otherwise, they were deemed proliferative. In addition, proliferative cells were designated G2/M if their G2/M score > S score, whereas cells were designated S if their S score > G2/M score.

### GO Term and KEGG Pathway Enrichment Analysis

Lists of genes were analyzed using the clusterProfiler R package (Yu et al., 2012)^3^, and the Benjamini–Hochberg procedure was used for multiple test corrections. Gene ontology (GO) (Pang et al., 2020)^4^ terms with a *P* value less than 0.05 and Kyoto Encyclopedia of Genes and Genomes (KEGG) (Kanehisa and Goto, 2000)^5^ terms with a *P* value less than 0.05 were considered as significantly enriched. GO term enrichment analysis of target genes of transcription factors (TFs) was performed using Metascape (Lu and Zhu, 2020)^6^, which was flexible for gene multiple functional analysis.

### Construction of Trajectory Using Variable Genes

Monocle (Trapnell et al., 2014)^7^ ordering was conducted for constructing single-cell pseudo-time of retinal cells using highly variable genes, which were identified by Monocle to sort cells in pseudo-time order with default parameters. “DDRTree” was applied to reduce dimensional space, and the minimum spanning tree on cells was plotted by the visualization functions “plot_cell_trajectory” or “plot_complex_cell_trajectory.” Branch Expression Analysis Modeling (BEAM) tests were performed on the first branch point of the cell lineage using all default parameters. Plot_genes_branched_pseudotime function was performed to plot a couple of genes for each lineage.

### Regulatory Network Construction

We downloaded human TF lists from AnimalTFDB (Zhang et al., 2012)^8^ as a TF reference and extracted TFs in marker gene lists of each cluster to construct the regulatory network. The extracted TFs were submitted to a STRING database (Szklarczyk et al., 2017)^9^ to infer regulatory networks based on known interaction relationships (supported by data from curated databases, experiments, and text-mining). TFs without any interactions with other proteins were removed from the networks.

### Construction of a Cellular Communication Network

The ligand–receptor interaction relationships were downloaded from the databases, namely, IUPHAR/BPS Guide to PHARMACOLOGY (Harding et al., 2018) and Ligand-Receptor Partners (DLRP) (Salwinski et al., 2004; Pavlicev et al., 2017). The average expression level of UMI number of 1 was used as a threshold. Ligands and receptors above this threshold were considered as expressed in the corresponding clusters (Pavlicev et al., 2017). The R package Circlize (Gu et al., 2014)^10^ was used to visualize the interactions.

### Construction of Cross-Tissue and Cross Cell-Type Correlation Network

To reduce noise, we averaged the expression of every 30 cells within clusters and then calculated the pairwise Pearson correlation between two dots based on their average expression profiles. Inter-dot relationships would be shown if their Pearson correlation was greater than 0.95. This correlation network was generated using Cytoscape (Shannon et al., 2003)^11^.

### Enriched Ontology Clusters

We first identified all statistically enriched terms. Accumulative hypergeometric p-values and enrichment factors were calculated and used for filtering. The remaining significant terms were then hierarchically clustered into a tree based on Kappa-statistical similarities among their gene memberships. Then, a 0.3 kappa score was applied as the threshold to cast the tree into term clusters. We then selected a subset of representative terms from this cluster and converted them into a network layout. More specifically, each term was represented by a circle node whose size was proportional to the number of input genes fall into that term and whose color represented its cluster identity. The network was visualized using Cytoscape (Shannon et al., 2003) (see text footnote 11) with a “force-directed” layout and with edge bundled for clarity. One term from each cluster was selected to have its term description shown as label.

### Protein–Protein Interaction Network

Molecular Complex Detection (MCODE) (Liao et al., 2020)^12^ algorithm was then applied to this network to identify neighborhoods where proteins were densely connected. Each MCODE network was assigned a unique color. GO enrichment analysis was applied to each MCODE network to assign “meanings” to the network component.

## Results

### Collection of the Trisomy 21 Retinal Tissue and Single Nucleus RNA-Seq

**FIGURE 1:**
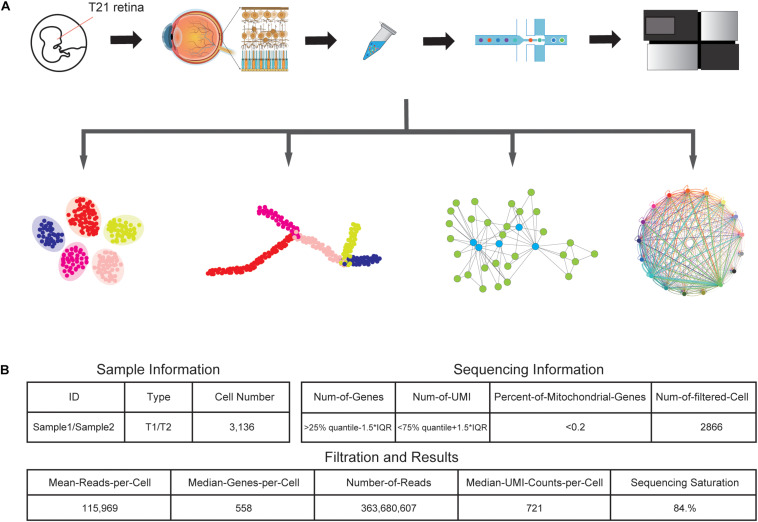
Generation of the T21 human fetal retina snRNA-seq dataset. **(A)** Schematic representation of the experimental workflow. Tissue collection, nuclei sample preparation, barcode attaching, snRNA-seq, and data analyzing processes were included in the experiment. **(B)** Summary statistics of sample and sequencing information, together with data size of snRNA-seq results.

We collected one retinal tissue from a trisomy 21 donor and dissociated the sample into a single-cell suspension without surface marker preselection, followed by snRNA-seq (Figure 1A). In total, around 6000 nuclei were subject to library construction. After sequencing, we obtained the single-cell transcriptome of 3,136 cells, with a mean coverage of 115,969 reads per cell. After filtering, a total of 2,866 cells were retained for subsequent analyses (Figure 1B).

### Cellular Heterogeneity in Retina Tissues

**FIGURE 2:**
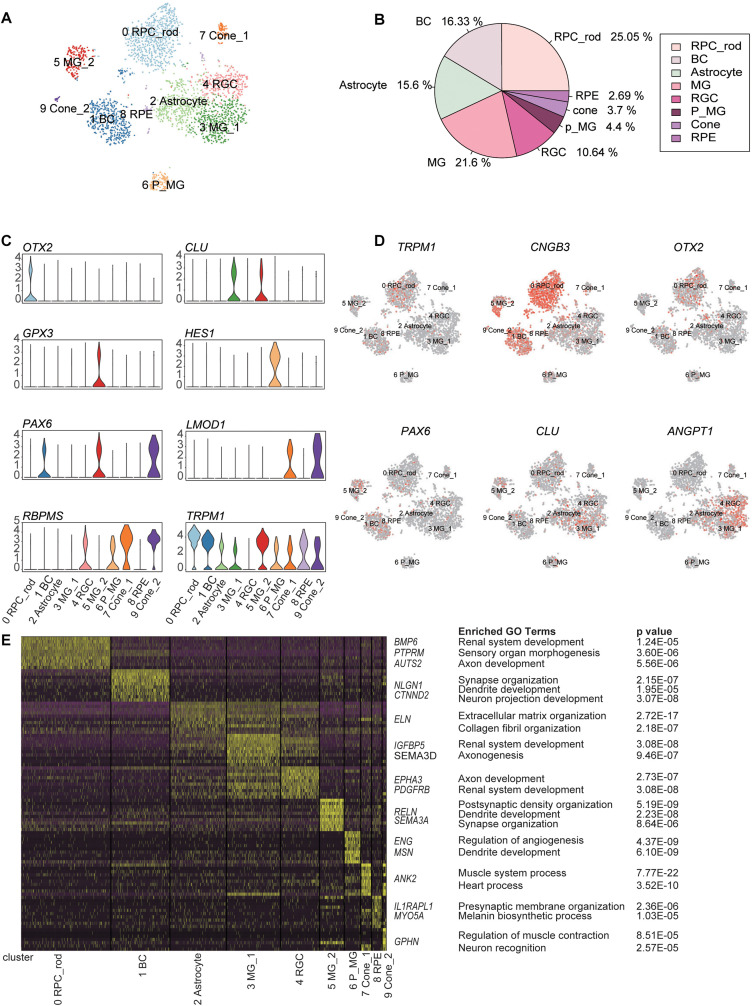
Classification of retina cell types according to distinct gene markers. **(A)** Two-dimension (2D) visualization of single-cell clusters acquired through t-Distributed Stochastic Neighbor Embedding (t-SNE). 2866 single-cell expression profiles were clustered into 10 retinal cell populations. Each dot represents a single cell, and clusters are color-coded. **(B)** Pie plot showing the proportions of eight cell types in the trisomy 21 data set. **(C)** Violin plots demonstrating expression levels of marker genes for cell clusters. **(D)** The t-Distributed Stochastic Neighbor Embedding (t-SNE) maps of retina single-cell data with colored cell based on the expression of marker genes for particular cell types. Gene expression levels are indicated by shades of red. **(E)** A gene expression heat map showing top differentially expressed genes for each cluster in 10 major clusters. Yellow corresponds to high expression level, while purple and black correspond to low expression level. Significant marker genes were listed on the right as well as enriched GO terms and corresponding probability values (*P* values).

Using the unsupervised clustering method, 2866 cells were classified into 10 major clusters, corresponding to seven cell types, including RPCs, BCs, rod PR cells, cone PR cells, retina ganglion cells (RGCs), MGs, and astrocytes (Figure 2A). This complex intrinsic composition of retina cells highlighted the necessity of single-cell technologies for dissecting the transcriptome signature in detail.

C0, C1, C2, C4, C7&C9, and C3&C5&C6 were annotated as retinal cells based on the specific expression of aforementioned cell-type markers (Supplementary Table 1) and GO term enrichment analysis of cluster-specific expressing genes (Supplementary Table 2) (Figures 2C–E and Supplementary Figures 2A,B). C0, expressing the RPC marker *OTX2*, was defined as RPCs. C1 was composed of BCs, according to the expression of *TRPM1*. C4 was considered to be RGCs due to the enrichment of *RBPMS*. C7 and C9 were identified as cone PRs, because of the specific expression of *LMOD1*.

C3, C5, and C6 expressed three different MG markers, respectively, namely, *CLU*, *GPX3*, and *HES1* (Liang et al., 2019). C6 displayed the characteristics of MGs with differentiation potential, based on the high expression of *HES1* (marker for RPCs) and the enrichment of development GO term such as dendrite development. The distinctions between C3 and C5 revealed two functional subtypes of MGs. Specifically, C3 was related to renal system development and axonogenesis GO terms, while C5 highly expressed *GPX3* and was associated with synapse organization term. Trajectory analysis further certified that these three clusters had a differential relationship and seemed to represent three differentiation stages of MGs (Lu et al., 2020). C2 and C8 were identified as astrocytes and RPE, respectively. C2 was characterized by the high expression of *ANGPT1* (Lu et al., 2020). C8, expressing RPE markers (*TYRP1* and *PMEL*) (Collin et al., 2019), was defined as RPE (Supplementary Figure 2B).

To explore the cellular compositions of T21 retina, we calculated the proportion of each retinal cell type (Figure 2B). The most abundant cell type was found to be rod PRs, followed by MGs and BCs. Cone PRs and RPE accounted for 3.7 and 2.69%, respectively. However, we noticed that two major retinal cell types (horizontal cells and amacrine cells) were undetected in our data. *LHX1*, acting as a horizontal cell-specific marker, was not detected in the gene expression profile matrix generated by Seurat, and the expression level of *LHX1* was 0. Meanwhile, the expression of amacrine cell-specific marker TFAP2A was very low, and the sporadic expression did not form one cell cluster (Supplementary Figure 3).

### Dissection of Retinal Neuron States Transition Through Developmental Trajectory Analysis

**FIGURE 3:**
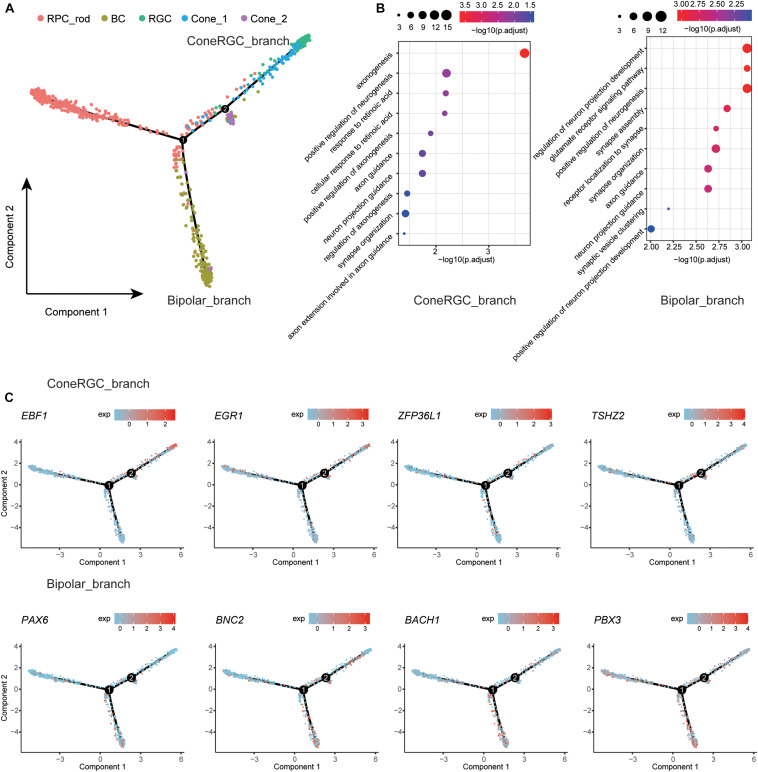
Developmental trajectory analysis of the retina neural cells state transition. **(A)** Single-cell trajectories by Monocle analysis showing development of the T21 human fetal retinal neural cells. **(B)** GO enrichment of ConeRGC_branch and Bipolar_branch upregulated genes. **(C)** Dynamic gene expression patterns of TFs for T21 retinal cell fate commitment.

To dissect the differentiation process among human retina, we first removed RPE and glial cells due to the irrelevance in the retinal developmental relationship (Tao and Zhang, 2014) and then merged remaining retina neural cell clusters to create a systematic landscape for the whole cell lineage using Monocle (Trapnell et al., 2014; Qiu et al., 2017a,b). Each cluster was ordered along with the pseudo-time (Figure 3A). To further investigate the molecular mechanism of cell types determination, we performed differential gene expression analysis along the constructed developmental path. Compared to cells located on RPC_branch, the upregulated genes were identified in cells located on ConeRGC_branch and Bipolar_branch, respectively. GO enrichment analysis suggested that those upregulated DEGs on ConeRGC_branch were enriched in biological functions associated with the regulation of axonogenesis and neurogenesis, while the upregulated DEGs on Bipolar_branch were mainly associated with the regulation of neuron projection development and glutamate receptor signaling pathway (Figure 3B and Supplementary Table 4). Intriguingly, a variety of transcription factor encoding genes, which have been previously reported to be closely associated with neuron development, were found to be upregulated in ConeRGC_branch (*EBF1*, *EGR1*, *ZFP36L1*, *TSHZ2*) and Bipolar_branch (*PAX6*, *BNC2*, *BACH1*, *PBX3*), respectively (Figure 3C and Supplementary Figure 4). *EBF1* has been reported to be required for the topographic projection of RGC (Jin and Xiang, 2011), and *PAX6* is required for the inhibition of photoreceptor fate during retinogenesis (Remez et al., 2017). Overall, our data revealed the dynamic expression patterns of TFs during the cell fate commitment of neural cells in T21 retina.

### Identification of Master Regulators Specifying PR Cells Development

Next, we reconstructed the developmental trajectories of PR cells according to cell differentiation status and cell subpopulations (Supplementary Figure 5A). In order to infer the role of TFs in cell status transition, we constructed the regulatory network using 13 differentially expressed TFs for RPCs and PR cells. We established the regulation network of these TFs by analyzing the genes co-expressed with TFs (Supplementary Figure 5B) and extracted the top 1000 links positively related to each TF. The results suggested that all TFs possessed strong regulatory function and that *MYRF*, *PRDM16*, *BNC2*, *NR2F1*, and *RARB* were the key regulators of the network since the extensive co-regulation with potential target genes was shown. Consistent with our functional annotation results, these key regulators played an important role in the process of retina formation and the light signal transition of PR (Supplementary Figure 5C).

### Construction of Müller Glial Cell Differentiation Trajectory

To characterize the MGs with differentiation potential, we extracted the MG clusters and constructed the trajectory with the unsupervised clustering gene (Supplementary Figure 5D). C6, with the specific expression of developmental markers, lay at the root state, while C3 and C5 lay at the two ends of development trajectory, respectively. We then compared the TF expression at three states and found that *MEIS1* was specifically expressed in C5, whose top 1000 target genes enrich GO terms of positive regulation of the neurological system process. *JUND* was highly expressed in C3 and C5 and correlated with the secretion and transport of the neurotransmitter. Interestingly, *CUX1* and *FOXP2* were expressed in C3 and C5, whose top 1000 target genes enrich amounts of immune regulatory function terms (Supplementary Figures 5E–H).

### Widespread Cross-Cell Type and Intercellular Communication Network

**FIGURE 4:**
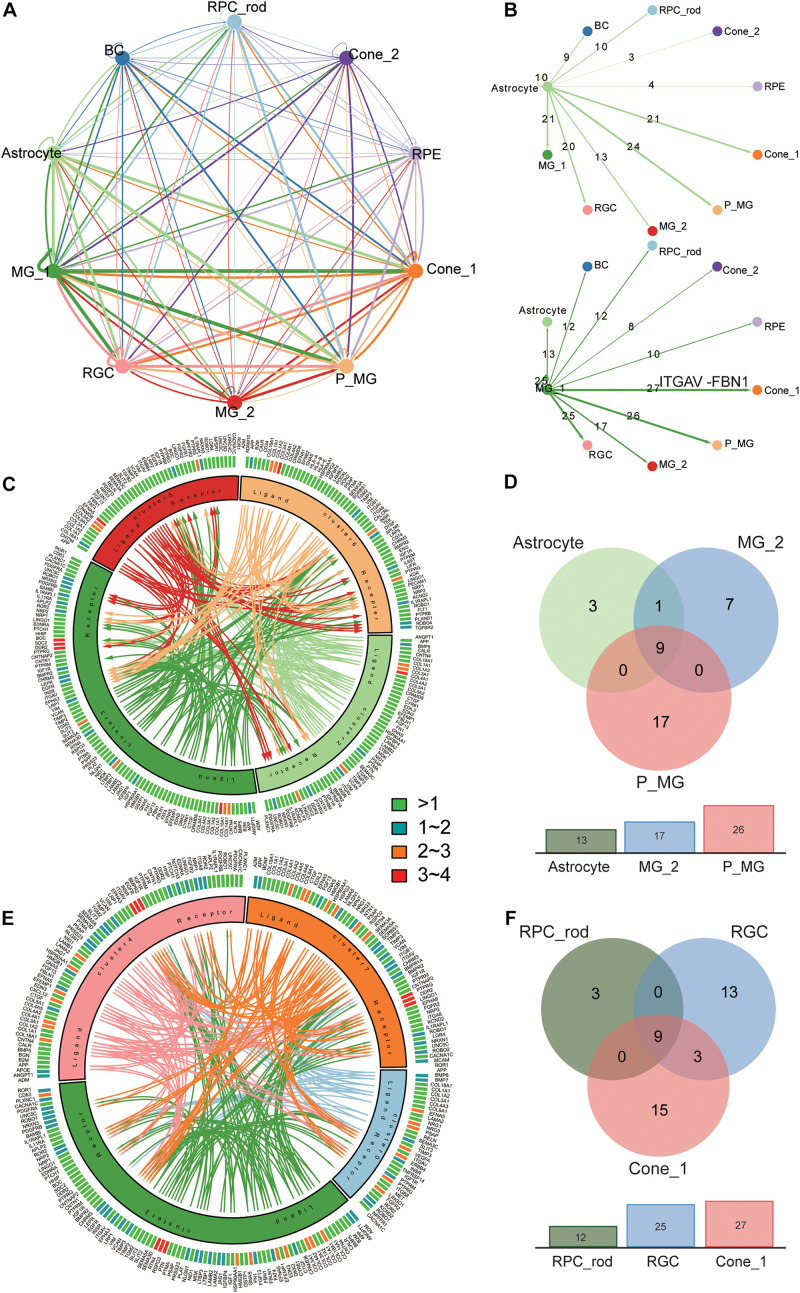
Analysis of the cross-cluster and intercellular communication network within T21 human fetal retina. **(A)** Summary of the communication network between 10 major clusters according to ligand–receptor interactions. Those populations expressing cognate receptors primed to receive a signal. **(B)** Detailed view of ligand–receptor interactions derived from Müller glial subtype (below) and astrocyte (above) ligands. Numbers of paired expressed receptors are shown for each pair of clusters. **(C)**. Visualization of the interactions between glial cell subtypes. **(D)** Comparison among communication pairs of three glial cell types regulated by MG subtype (Cluster 3), with the total number of them shown by the corresponding bar plots below. **(E)** Visualization of the interactions between MG subtype (Cluster 3) and other retinal neural cells (Cluster 0, 4, 7). **(F)** Comparison among communication pairs of three retinal neural cell types regulated by MG subtype (Cluster 3), with the total number of them shown by the corresponding bar plots below.

Based on the identification of several different MG types and astrocytes and their intense communication with other retinal cell types found in the previous study (Nagashima et al., 2017), we moved on to explore the cross-cluster and intercellular communication network within the retina. MGs play a crucial role in supporting the development, survival, and information processing of the neuron (Bringmann et al., 2006). The cellular interactions were inferred using the public ligand–receptor database (see section “Materials and Methods”). Although we did not take anatomical barriers into consideration, the expression patterns of ligand–receptor pairs in the networks revealed dense cross-cluster and intercellular communication networks (Figure 4A and Supplementary Table 5). In the cross-cluster network, 56 ligands and 36 receptors were expressed within C3 and the most frequent interaction was observed in the subtype of MGs. Moreover, C6 and C7 clusters received the most interactions. In detail, the interaction between clusters was characterized by the number of ligands and receptors (Figure 4B and Supplementary Figures 6A–H). To further explore the role of MGs in supporting neural cells, we extracted the communication pairs between MGs and other retinal neural cells to compare the shared and cell-type specific interactions (Figures 4C,E and Supplementary Figure 6I). We found the ITGAV receptor (expressed in all retinal neural cell types) together with ligand FBN1 to be the most frequent interaction pair in the MG signaling network (Figures 4B,D,F and Supplementary Table 6). The FBN1-ITGAV pair is shown to be involved in mediating R–G–D-dependent cell adhesion (Jovanovic et al., 2007). Likewise, the widely expressed receptor ROBO1 appeared simultaneously with its cognate counterpart SLIT2, and this interaction pair has been shown to hold a crucial role in the regulation of commissural axon pathfinding (Ricano-Cornejo et al., 2011). Both *LRP1* and *CD74*, expressed in C4 and C6, respectively, might bind to the APP protein expressed in C3, which are associated with different signaling circuits regarding the formation and function of retina (Matsuda et al., 2009). Moreover, the Müller glia-expressing ligand CTGF has previously been shown to regulate nervous system development through the ERBB4 (specifically expressed in C0) signaling pathway (Haskins et al., 2014). As for the communications between MGs and all other glial cell types, it was found that *DDR2* was specifically expressed in astrocytes and interacted with *COL3A1* and *COL1A1*, which are involved in the maintenance of immune homeostasis in retina (Vecino et al., 2016).

### Dissection of Single-Cell Transcriptome of Trisomy 21 Retina

**FIGURE 5:**
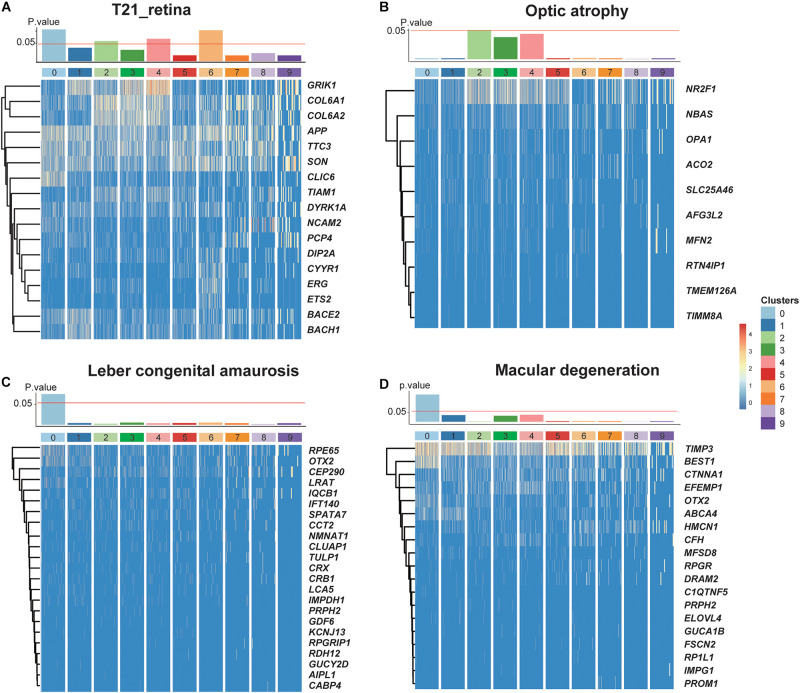
The dosage effects of the redundant chromosome 21 on T21 retina. **(A–D)** *P* values of enrichment of each cluster about trisomy 21 dosage genes and three retinal diseases (optic atrophy, Leber congenital amaurosis, and Macular degeneration) related genes, together with heat maps of their corresponding significantly expressing genes.

Given that our data came from a T21 donor, we investigated the dosage effect of supernumerary chromosome 21 on the retina. By analyzing the expression profiles of genes located on chromosome 21, we performed a hypergeometric enrichment test (see section “Materials and Methods”) and found that the DEGs in RPCs and one subtype of MGs were significantly enriched in chromosome 21 gene expression, indicating the association between the influence of supernumerary chromosome 21 and the specific cell types (Figure 5A). For instance, CLIC6 and NCAM2 specifically expressed in RPCs were both encoded by chromosome 21, as well as ERG and ETS2 specifically expressed in the Müller glial subtypes with the developmental potential (Supplementary Figure 7). Moreover, the retinal diseases with the associated gene lists were also related to several cell types (Figures 5B–D). Leber congenital amaurosis and macular degeneration were enriched in C0, which specifically expressed *RPE65*, *OTX2*, *LRAT*, and *BEST1* (Henderson et al., 2009; Hosono et al., 2018). Optic atrophy was enriched in astrocytes specifically expressing *NR2F1* and *AFG3L2* (Bosch et al., 2014; Charif et al., 2015).

## Discussion

In this study, we conducted snRNA-seq of the retina from a trisomy 21 fetus to dissect the heterogeneity of the retina, establishing the first retinal cell atlas under T21 conditions. We also studied the influence of supernumerary chromosome 21 in the development deficiency and gene expression dysregulation of trisomic retina.

In total, seven major cell types and several subtypes within each cell type were identified, including RPCs, BCs, rod PRs, cone PRs, RGCs, MGs, and astrocytes. It was noteworthy that two retinal neuronal cells, namely, horizontal cells and amacrine cells, were not detected in T21 retina. In contrast, both types of retinal cells have been identified in the single-cell transcriptome studies of human disomic fetal retina (Lu et al., 2020) and other mammalian retina (Macosko et al., 2015; Shekhar et al., 2016). Horizontal cells are a kind of multipolar neurons. In the outer plexiform layer of the retina, horizontal cells receive the input signal of photoreceptors and regulate the information transmission of photoreceptors to bipolar cells through a feedback mechanism. In addition, amacrine cells are interneurons of the retina. The dendrite bifurcations of amacrine cells extend to the inner plexiform layer, where they are in contact with RGCs and BCs (Masland, 2001). The function of amacrine cells is mainly responsible for the information output from bipolar cells. The damage or loss of horizontal cells and amacrine cells directly causes the abnormality of the visual conduction pathway. Due to the prevalence of developmental disorders in T21 patients (Bhattacharyya and Svendsen, 2003), combined with the above findings, we speculated that the absence of these two cell types in trisomic retina might be closely related to the delay of retina development, and this cell development deficiency might contribute to the visual impairment in T21 patients.

Meanwhile, we analyzed the proportion of various retinal cell types of the T21 fetus in detail. Our analysis showed that the most common cell type was rod PRs, accounting for 25%, and other cell types (such as MGs and BCs) also accounted for a certain proportion. In contrast, the cone PRs presented a lower proportion of cells in T21 retina. These results were significantly different from that of disomic fetus retina (Lu et al., 2020), further confirming the abnormal cell compositions of trisomic retina. The relationship between the specificity of retinal cell compositions and the pathological features and clinical phenotypes of the eye in T21 patients deserves further study in the future.

In order to fully understand the differentiation process of T21 retina, the retina neural cell clusters were extracted for pseudo-time analysis. The differentially expressed TFs were found along the constructed developmental path, which were closely related to the cell fate commitment of retina neural cells. We further constructed the developmental trajectories of PR cells and MGs and analyzed the key regulatory factors in the development process. In PR cells, several key TFs related to cell differentiation and development were demonstrated. Among these, *PRDM16* (Shimada et al., 2017), *BNC2* (Patterson and Parichy, 2013), and *RARB* (Gely-Pernot et al., 2015) are all involved in the positive regulation of cell differentiation and development. The function of MYRF (Yin et al., 2020) protein mainly focuses on the regulation of myelination in the nervous system. The transcription factor *NR2F2* performs the crucial role in early differentiation of human embryonic stem cells by regulating the activation of neural genes (Rosa and Brivanlou, 2011).

To characterize the certain MGs with differentiating potency, the MG clusters were extracted to construct the developmental trajectory. Consistent with the cell annotation results, C6, which specifically expressed the progenitor cell marker *HES1*, lay at the root state, while two functional subtypes C3 and C5 were at the opposite ends of development trajectory, respectively. Subsequently, we analyzed the specific expression of transcription factors in three states. The results showed that there were co-expressed genes in the two functional cell subtypes C3 and C5, such as *JUND*, *CUX1*, and *FOXP2*. According to the enrichment analysis of the GO term, these genes were not only closely related to the secretion and transportation of neurotransmitters but also involved in the regulation of immune response, suggesting the multiple supportive and regulatory function of MGs during human retina development.

Next, we constructed a detailed communication network between MGs and other retinal neural cells, and the ligand–receptor pairs related to nervous system development and retina formation were identified. As expected, the interaction pairs found in astrocytes were involved in immune system regulation. The densely connected communication network revealed the extensive interactions within retinal cells, plenty of which deserve thorough studies in the future. In particular, the most frequent interaction in the subtypes of MGs was identified, namely, the FBN1–ITGAV pair. It has been reported that FBN1 mediates cell adhesion via interacting with its major receptor ITGAV, which is a member of the integrin family (Jovanovic et al., 2007). Meanwhile, ITGAV can regulate the differentiation of human adipose-derived stem cells by affecting the interactions between cells and the extracellular matrix (Morandi et al., 2016). We speculated that the FBN1–ITGAV pair might play an important role in the directional differentiation of MGs with differentiating potency. Further researches and experimental verifications are needed to confirm this hypothesis.

In clinical research, it is found that there are retinal abnormalities in T21 individuals, which can be manifested as abnormal retinal thickness (da Cunha and Moreira, 1996; Laguna et al., 2013). At the same time, the visual functions of T21 patients are often low (O’Brien et al., 2015). Our results confirmed the abnormality of retinal cell composition at the single-cell level. However, combined with previous studies, the pathological mechanism of retinopathy in T21 individuals has not been fully elucidated. To explore the effect of supernumerary chromosome 21 on the T21 retina, we analyzed the expression profiles of genes located on chromosome 21. The result showed that the DEGs in two cell types, namely, RPCs and one subtype of MGs, were significantly enriched in chromosome 21. We hypothesized that RPCs and MGs were closely related to retinal abnormalities caused by supernumerary chromosome 21 in Down syndrome. These two cell types with differentiation potential perform important functions during the development of retina, which are the key factors to maintain the normal structure and function of retina. Also, these DEGs in RPCs and one subtype of MGs have been reported to be associated with cell differentiation and development. The neural cell adhesion molecule NCAM2 specifically expressed in RPCs can regulate the differentiation of dendrite and axon (Parcerisas et al., 2020), and mutation of the *NCAM2* gene causes neurodevelopmental disorders (Petit et al., 2015). *ERG* specifically expressed in the MG subtype is considered as the transcription factor ETS-related gene and plays a critical role in B cell development and hematopoietic stem cell maintenance (Sondergaard et al., 2019). Moreover, CLIC6 was specifically expressed in RPCs and also encoded by chromosome 21. Although the regulatory role of CLIC6 in cell differentiation has not been reported, CLIC5, which belongs to the same chloride intracellular channel family as CLIC6, has been proven to be involved in the proliferation and differentiation of myoblasts (Li et al., 2010). According to the above findings, the pathological changes of the retina in trisomy 21 may be caused by the abnormal differentiation of RPCs and MGs, resulting in retinal development deficiency and eventually visual impairment. In this pathological process, these DEGs described above and the related signaling pathway of cell differentiation are likely to be the key factors in regulating the differentiation of RPCs and MGs. Furthermore, we analyzed the relationship between common retina diseases and trisomic retinal cell types based on the expression of disease-related genes. Interestingly, Leber congenital amaurosis and macular degeneration were significantly enriched in C0 (RPCs), further demonstrating the importance of RPCs in the pathological process of trisomy 21.

## Conclusion

In conclusion, we firstly described the single-cell transcriptome profile for human T21 retina to identify the cellular compositions in T21 retina, further demonstrating the difference of cellular types between T21 retina and disomic retina. Our data revealed the RPC subtypes with the potential different retinal cell-type commitments and MGs with differentiation potency. The extensive communication networks between cellular types were also confirmed. Further detailed analysis of cell components of human T21 retina based on snRNA-seq will be helpful to understanding the influence of supernumerary chromosome 21 on the development of retina.

## Data Availability Statement

The original contributions presented in the study are included in the article/Supplementary Material, further inquiries can be directed to the corresponding author.

## Code Availability

Custom codes for this project have been uploaded to GitHub and could be freely accessible from the following link: https://github.com/chaichaochao/T21.

## Ethics Statement

The studies involving human participants were reviewed and approved by Medical Ethics Committee of Shiyan Taihe Hospital (201813). Written informed consent to participate in this study was provided by the participants’ legal guardian/next of kin.

## Author Contributions

J-HW and F-YH conceived and designed this study. J-HW, F-YH, and S-HZ coordinated and collected the human T21 retina, analyzed the sequencing data, and wrote and revised the manuscript. F-YH, F-JG, and PX conducted the experiments of retina sampling. All authors read and approved the final manuscript.

## Conflict of Interest

The authors declare that the research was conducted in the absence of any commercial or financial relationships that could be construed as a potential conflict of interest.

## Footnotes

1 https://satijalab.org/seurat/

2 http://www.proteinatlas.org/

3 http://bioconductor.org/packages/release/bioc/html/clusterProfiler.html

4 http://geneontology.org/

5 https://www.genome.jp/kegg/

6 http://metascape.org/gp/index.html

7 http://cole-trapnell-lab.github.io/monocle-release/

8 http://bioinfo.life.hust.edu.cn/AnimalTFDB/

9 http://string-db.org/cgi/input.pl

10 https://cran.r-project.org/web/packages/circlize/

11 https://cytoscape.org/

12 http://apps.cytoscape.org/apps/mcode

## Supplementary Material

Supplementary Figure 1 **(A)** Visualization of cell cycles of clusters by pie chart and scatter plot. Clusters are distinguished by colors. **(B)** Presentation of the gene expression number (above), UMI number (intermediate), and the mitochondrial genes proportion (below) in clusters by violin plots. **(C)** Dot plots demonstrating the correlations of the UMI and mitochondrial genes (above) as well as the UMI and genes (below). **(D)** Venn plot showing housekeeping gene expression in our dataset.

Supplementary Figure 2 **(A)** Box plot of cell-type marker genes specifically expressed in clusters. **(B)** Expression of several melanin markers in clusters. Black-color points represent marker expression cells.

Supplementary Figure 3 Boxplot showing the expression level of *TFAP2A* in cell clusters.

Supplementary Figure 4 Boxplot showing the expression levels of four genes (*PAX6*, *BNC2*, *BACH1*, and *PBX3*) in RPC_rod_branch, ConeRGC_branch, and Bipolar_branch.

Supplementary Figure 5 **(A)** Analysis of development trajectories of cone cells. The different colors in upper panel mean different cell differentiation states predicted by Monocle, while colors in lower panel mean different cell types. **(B)** Regulatory network of 13 differentially expressed TFs for RPCs and PR cells in cone cells. **(C)** Go term enrichment analysis of the differentially expressing TFs (*MYRF*, *PRDM16*, *BNC2*, *NR2F1*, and *RARB*) in cone subtypes. **(D)** The differentiation trajectories of the three subpopulations of MGs. Three different colors represent the three subpopulations of MGs. **(E–H)** Enrichment analysis of specific transcriptional regulators in different differentiated branches of MGs.

Supplementary Figure 6 **(A–H)** Interactions between clusters are shown according to ligand-receptor pairs derived from ligands of each cluster. Numbers of ligand-receptor pairs are marked in plots. **(I)** The detail visualization of the interaction between clusters 2/3/4/6/7.

Supplementary Figure 7 Violin plots of genes on chromosome 21 specifically expressed in clusters.

Supplementary Table 1 Retina cell type markers collected from literatures.

Supplementary Table 2 Cluster DEGs and GO enrichment.

Supplementary Table 3 Cell cycle markers collected from literatures.

Supplementary Table 4 Up-regulated genes and enriched GO terms in ConeRGC_branch and Bipolar_branch cells compared to RPC_branch.

Supplementary Table 5 The summary of ligand-receptor pairs in cross-cluster and intercellular communication networks.

Supplementary Table 6 List of receptor-ligand pairs present in MGs and other clusters.
