# Supplementary figures and images for: Cell Development Deficiency and Gene Expression Dysregulation of Trisomy 21 Retina Revealed by Single-Nucleus RNA Sequencing
Source: Front Bioeng Biotechnol. 2020 Sep 23;8:564057. doi: 10.3389/fbioe.2020.564057 (PMC7538860; doi:10.3389/fbioe.2020.564057)

Figure S1

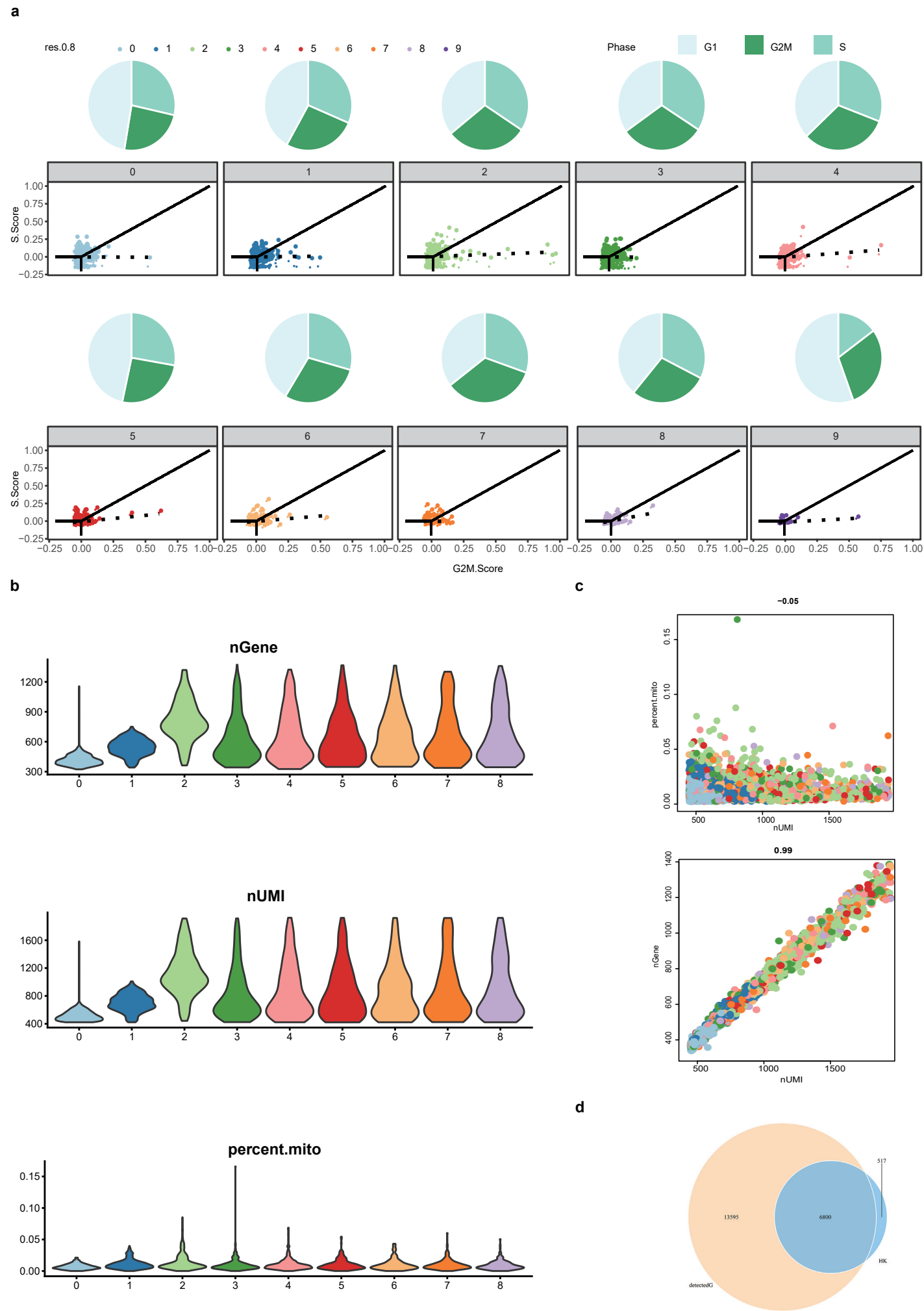

Supplement: Supplementary Figure 1 — (A) Visualization of cell cycles of clusters by pie chart and scatter plot. Clusters are distinguished by colors. (B) Presentation of the gene expression number (above), UMI number (intermediate), and the mitochondrial genes proportion (below) in clusters by violin plots. (C) Dot plots demonstrating the correlations of the UMI and mitochondrial genes (above) as well as the UMI and genes (below). (D) Venn plot showing housekeeping gene expression in our dataset. [file Image_1.pdf]

Figure S2

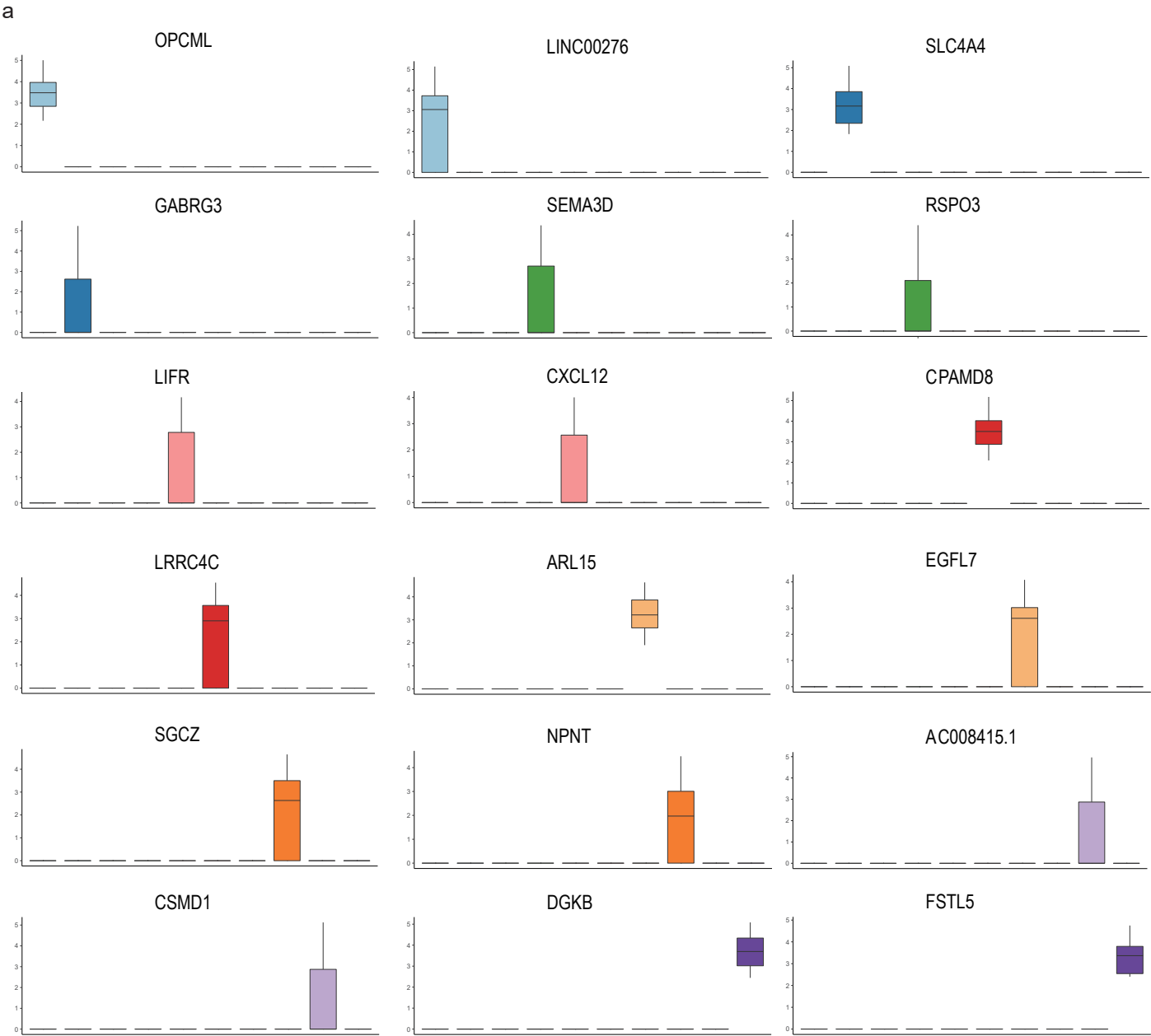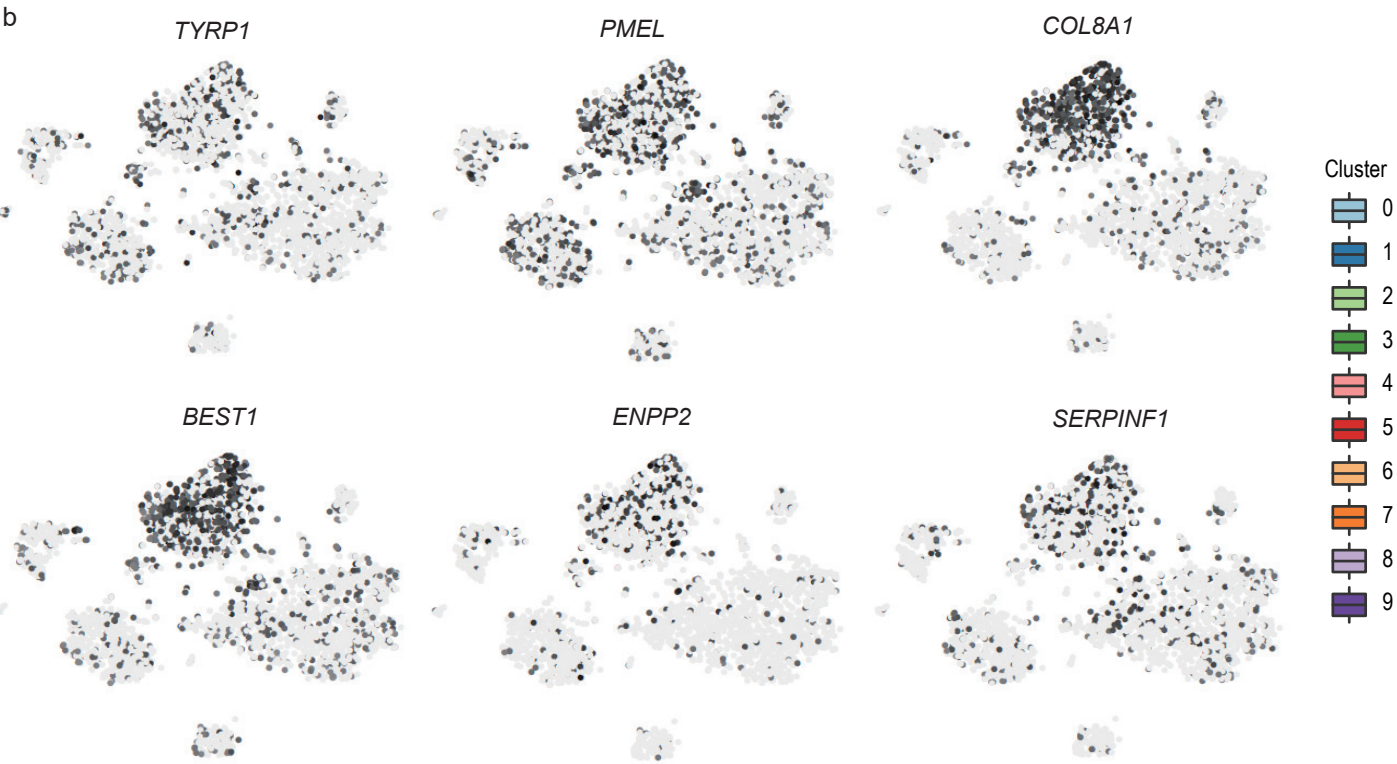

Supplement: Supplementary Figure 2 — (A) Box plot of cell-type marker genes specifically expressed in clusters. (B) Expression of several melanin markers in clusters. Black-color points represent marker expression cells. [file Image_2.pdf]

**Figure S3**

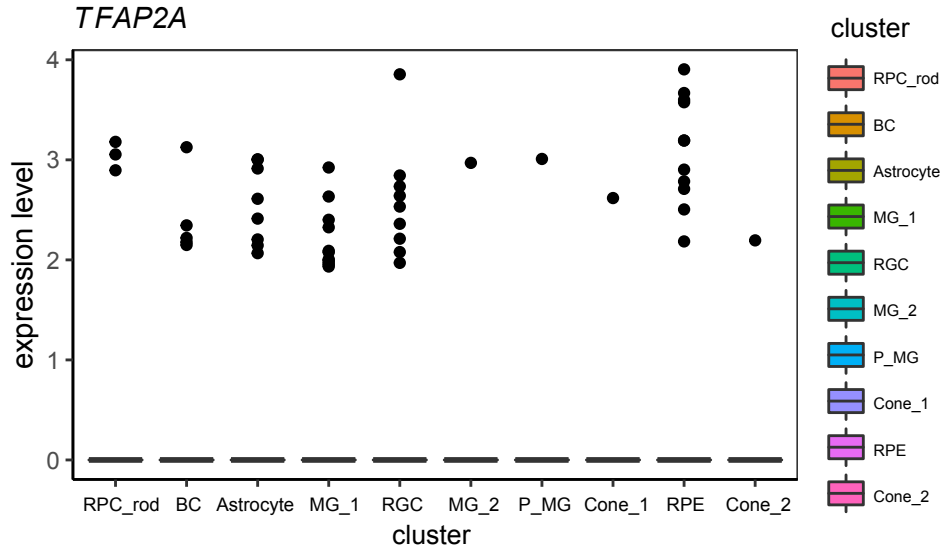

Supplement: Supplementary Figure 3 — Boxplot showing the expression level of TFAP2A in cell clusters. [file Image_3.pdf]

**Figure S4**

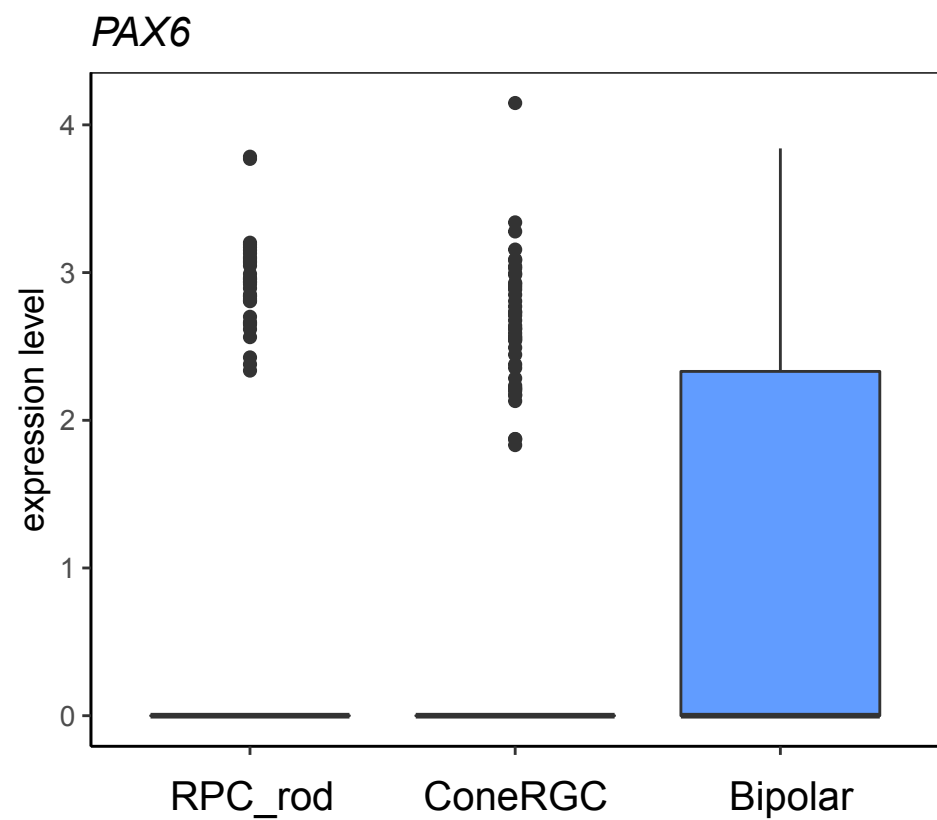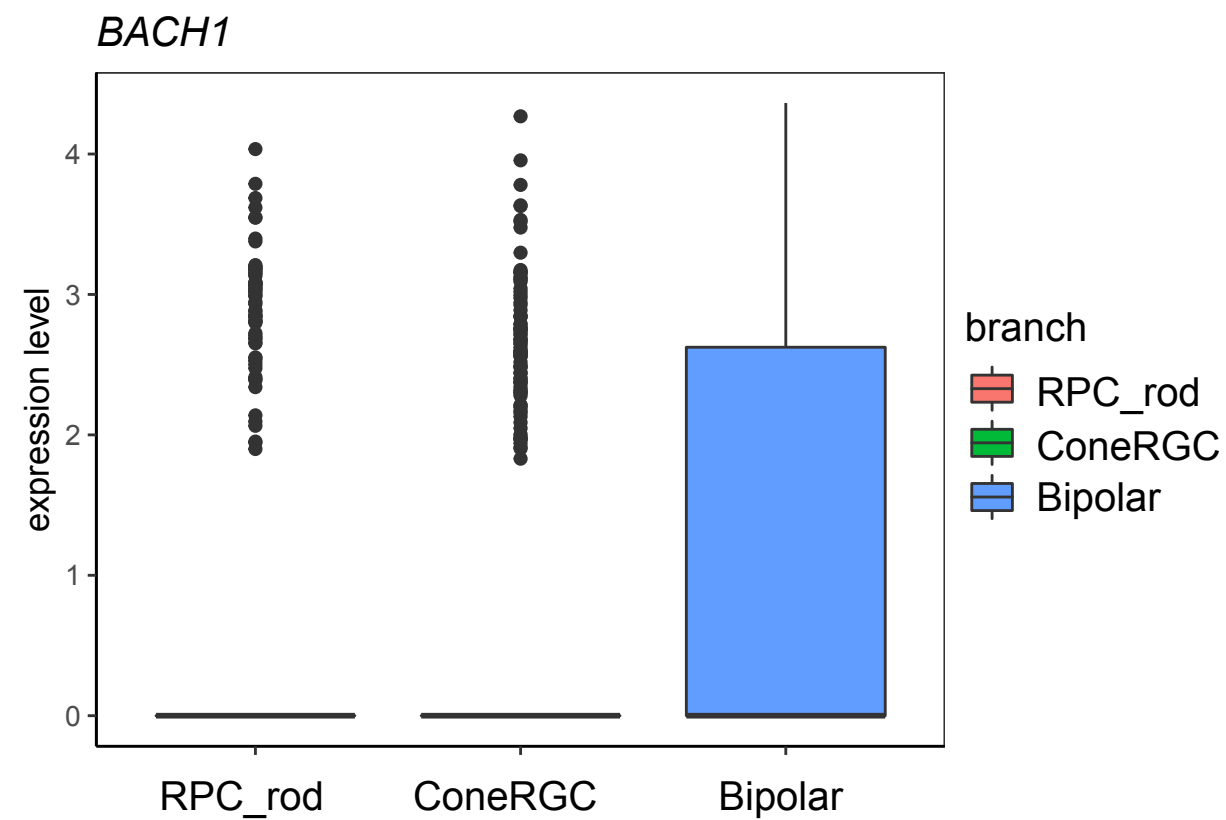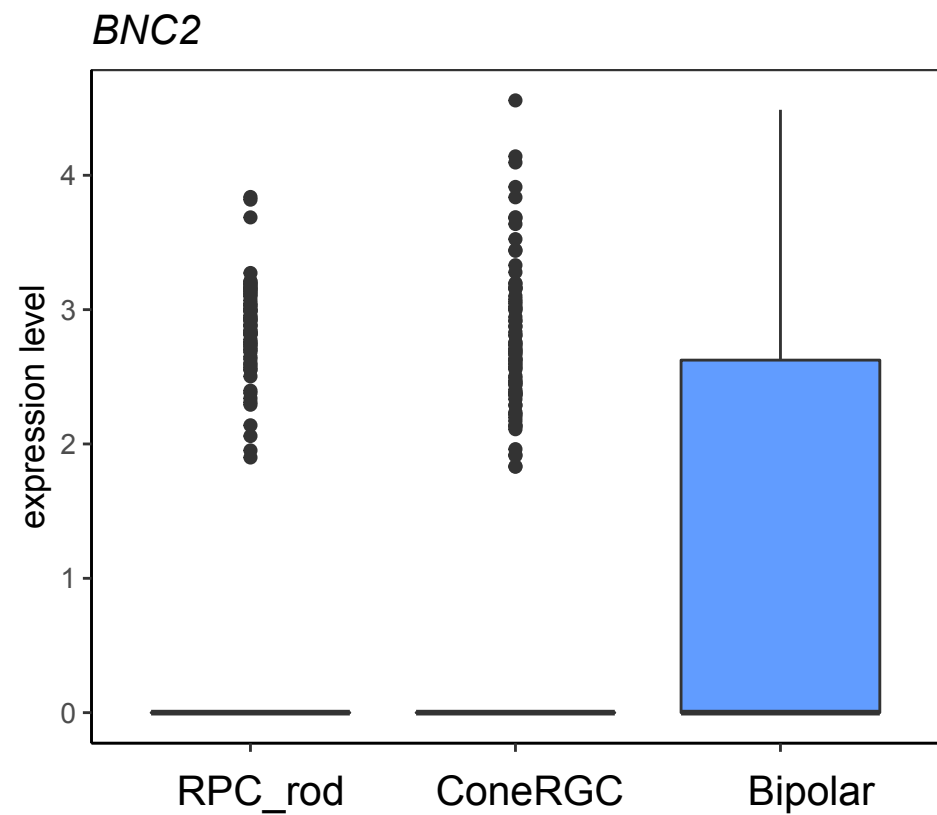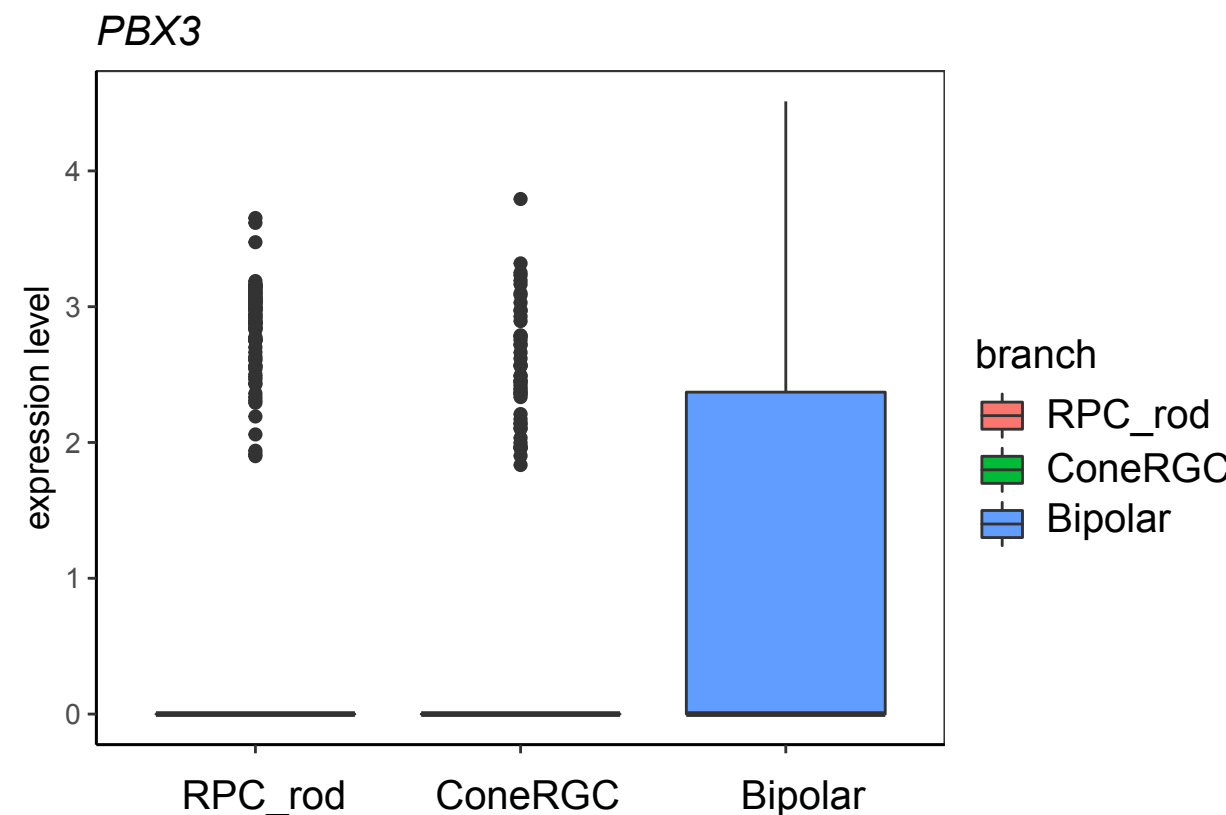

Supplement: Supplementary Figure 4 — Boxplot showing the expression levels of four genes (PAX6, BNC2, BACH1, and PBX3) in RPC_rod_branch, ConeRGC_branch, and Bipolar_branch. [file Image_4.pdf]

Figure S5

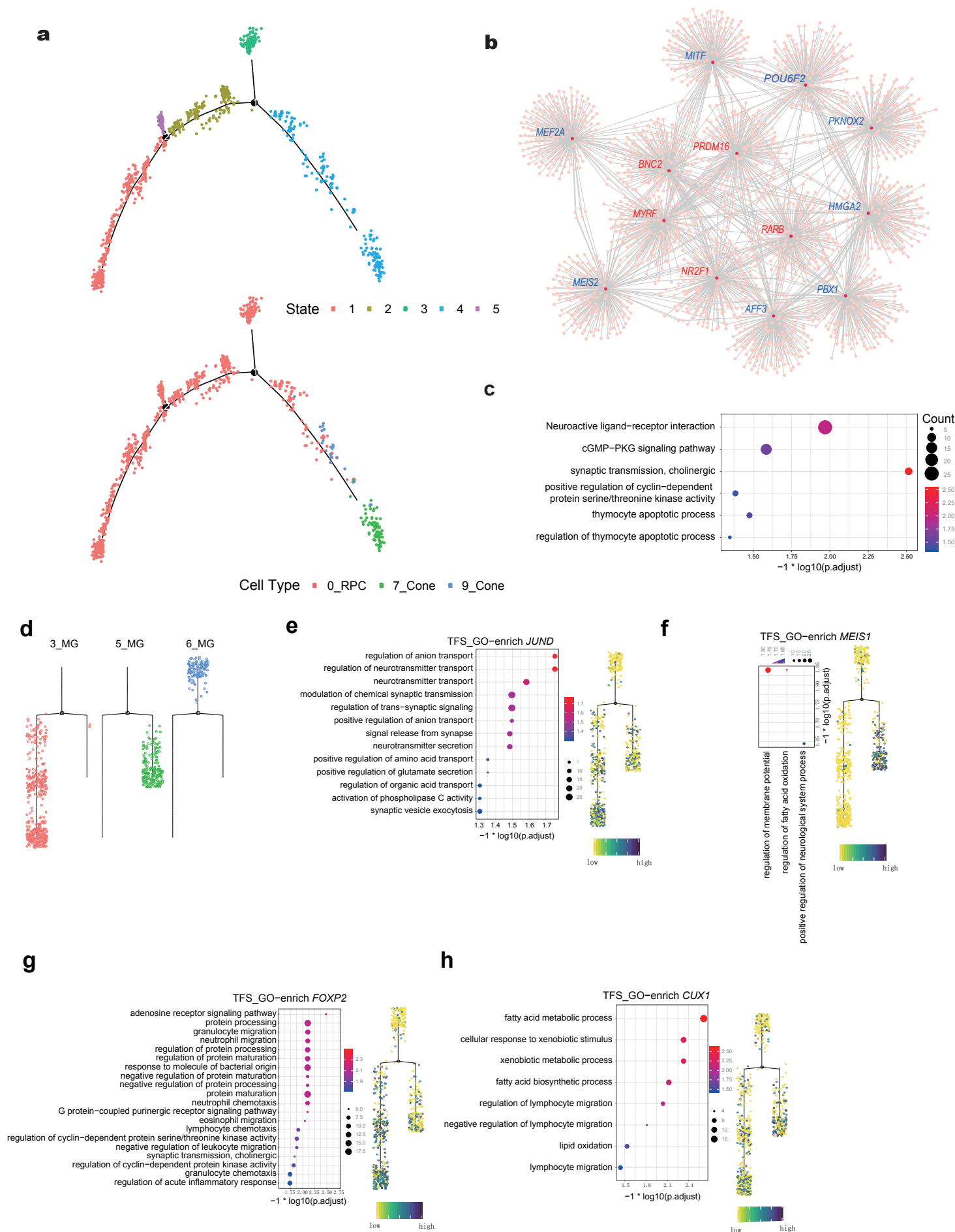

Supplement: Supplementary Figure 5 — (A) Analysis of development trajectories of cone cells. The different colors in upper panel mean different cell differentiation states predicted by Monocle, while colors in lower panel mean different cell types. (B) Regulatory network of 13 differentially expressed TFs for RPCs and PR cells in cone cells. (C) Go term enrichment analysis of the differentially expressing TFs (MYRF, PRDM16, BNC2, NR2F1, and RARB) in cone subtypes. (D) The differentiation trajectories of the three subpopulations of MGs. Three different colors represent the three subpopulations of MGs. (E–H) Enrichment analysis of specific transcriptional regulators in different differentiated branches of MGs. [file Image_5.pdf]

Figure S6

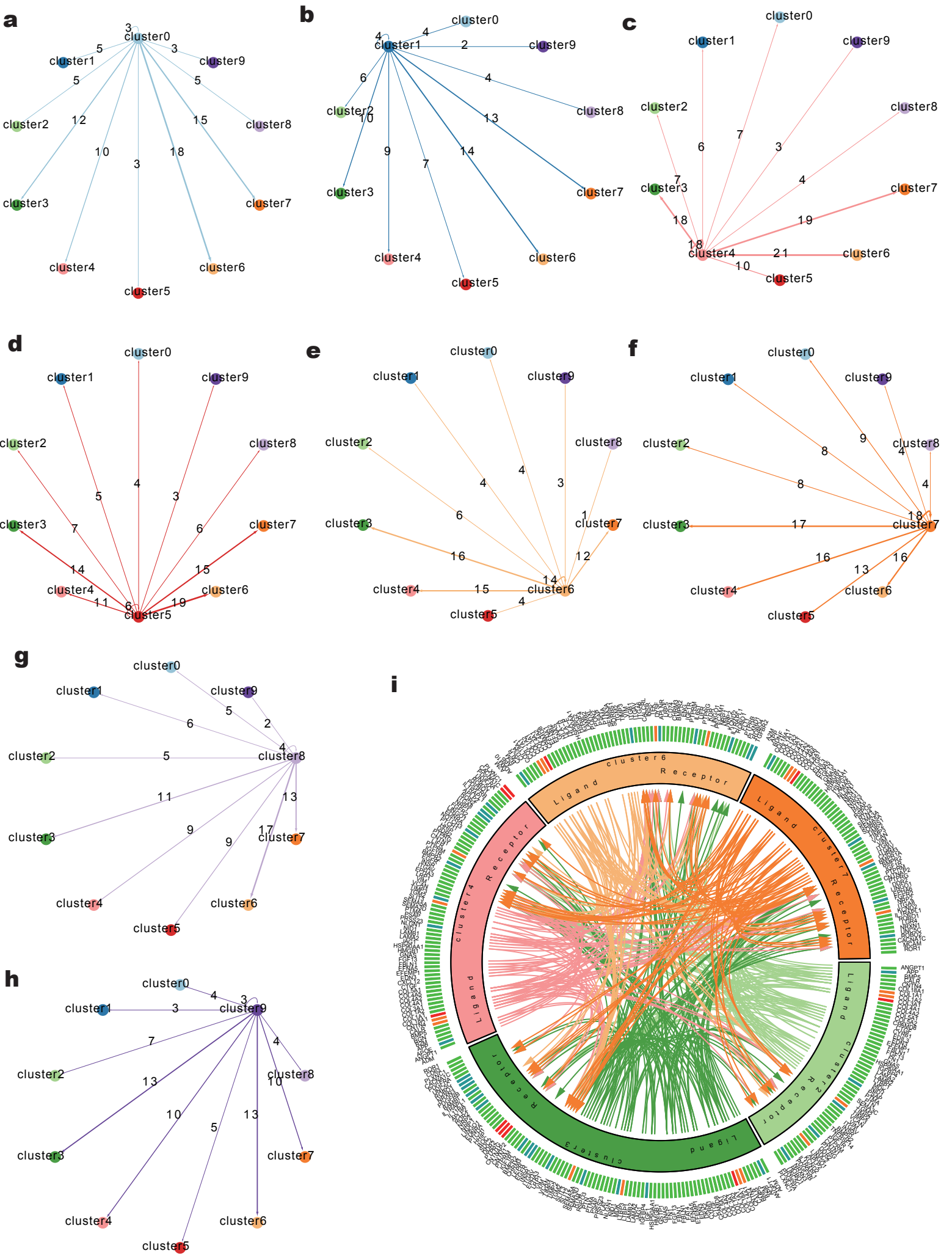

Supplement: Supplementary Figure 6 — (A–H) Interactions between clusters are shown according to ligand-receptor pairs derived from ligands of each cluster. Numbers of ligand-receptor pairs are marked in plots. (I) The detail visualization of the interaction between clusters 2/3/4/6/7. [file Image_6.pdf]

Figure S7

*CLIC6*

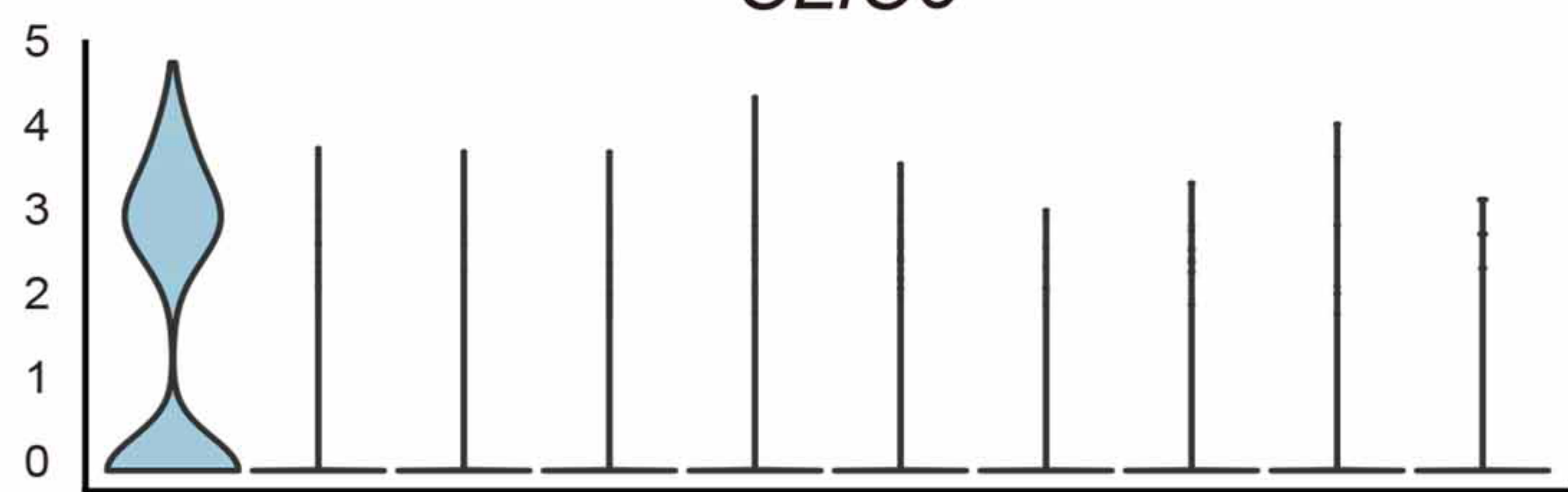

*CYYR1*

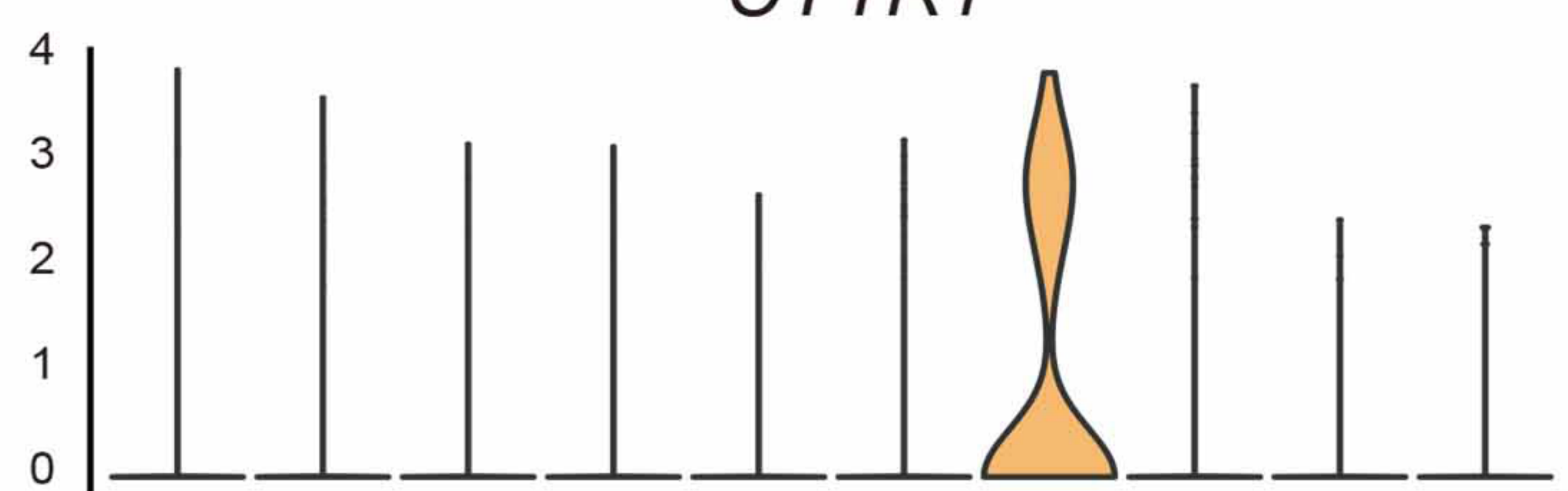

*NCAM2*

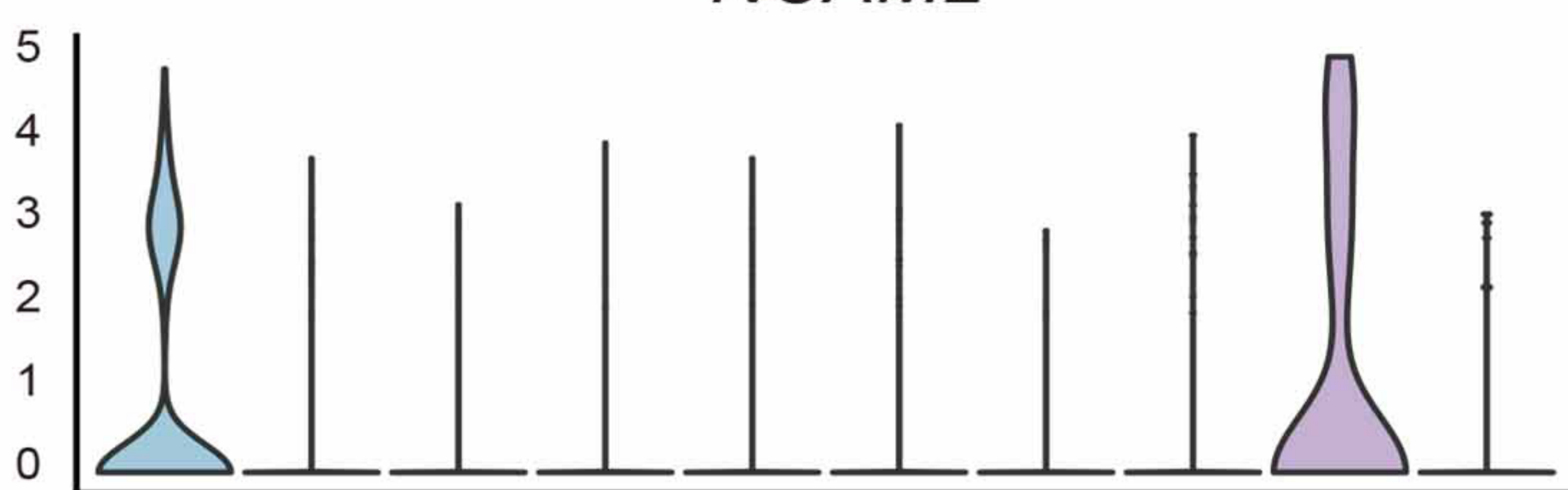

*ERG*

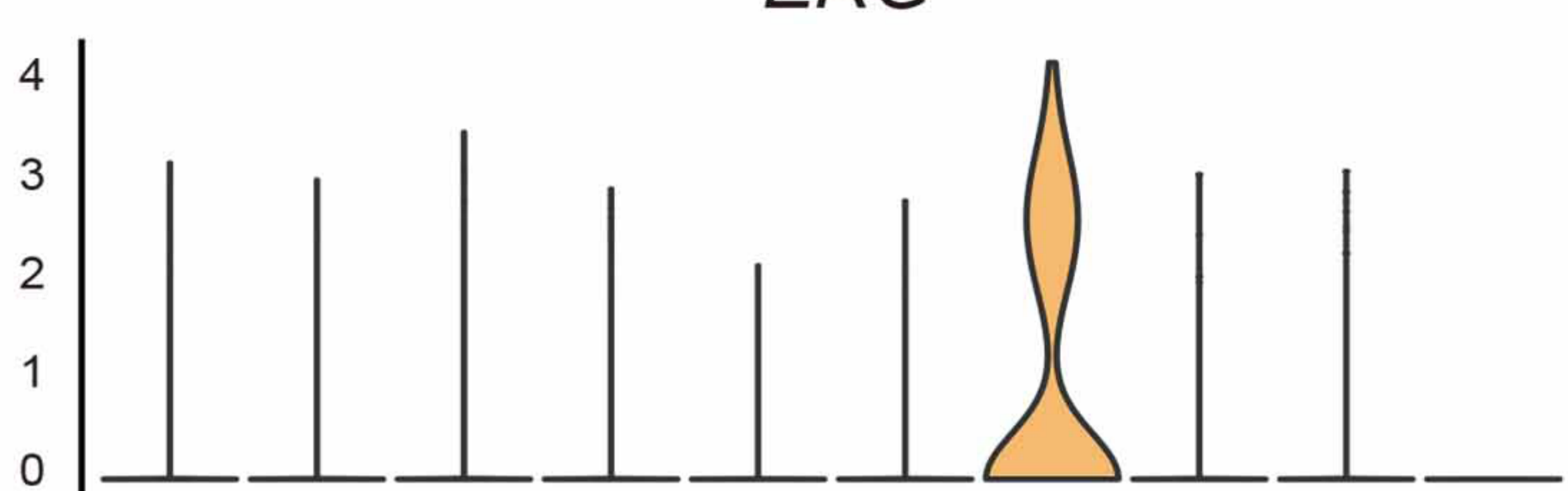

*BACE2*

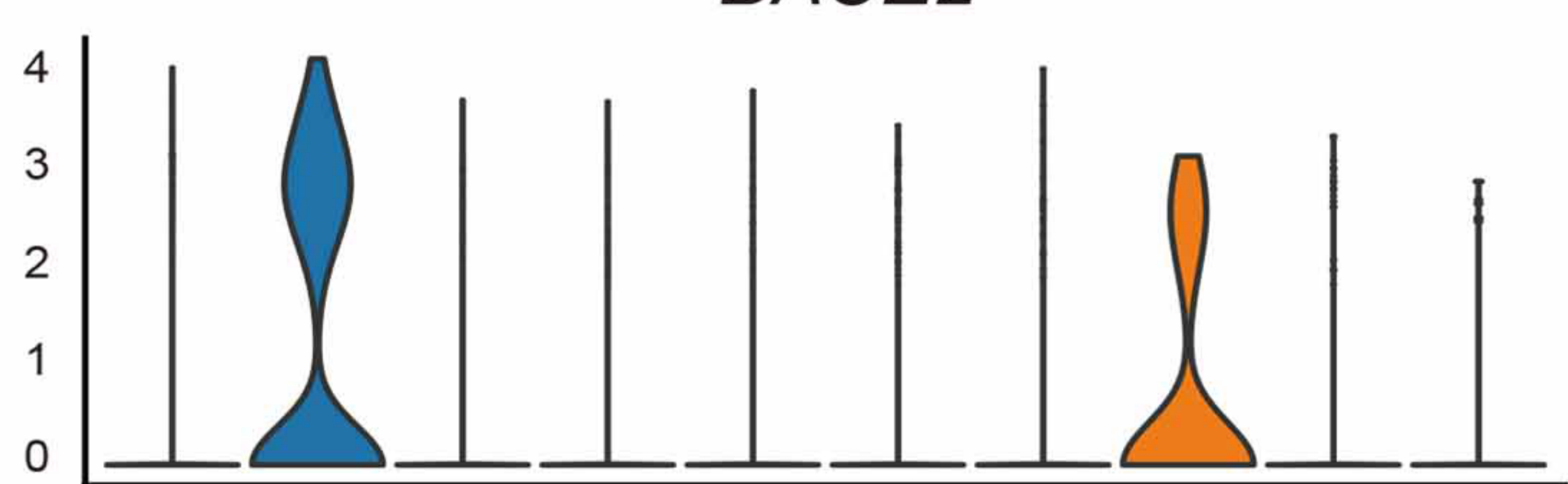

*ETS2*

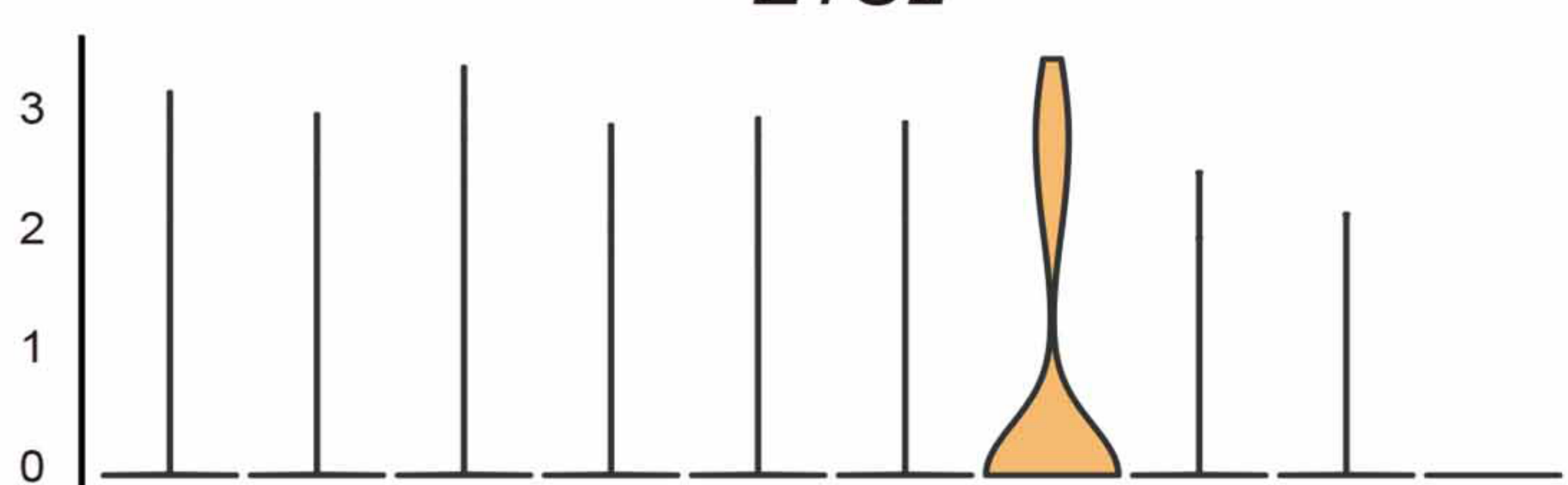

*BACH1*

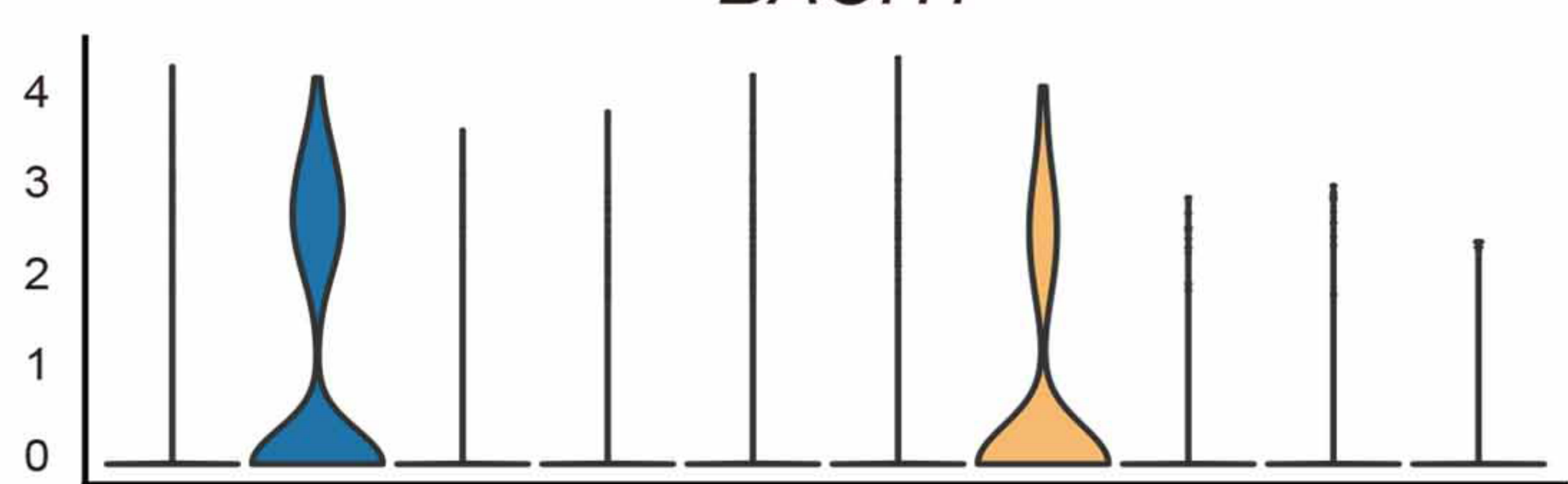

*DIP2A*

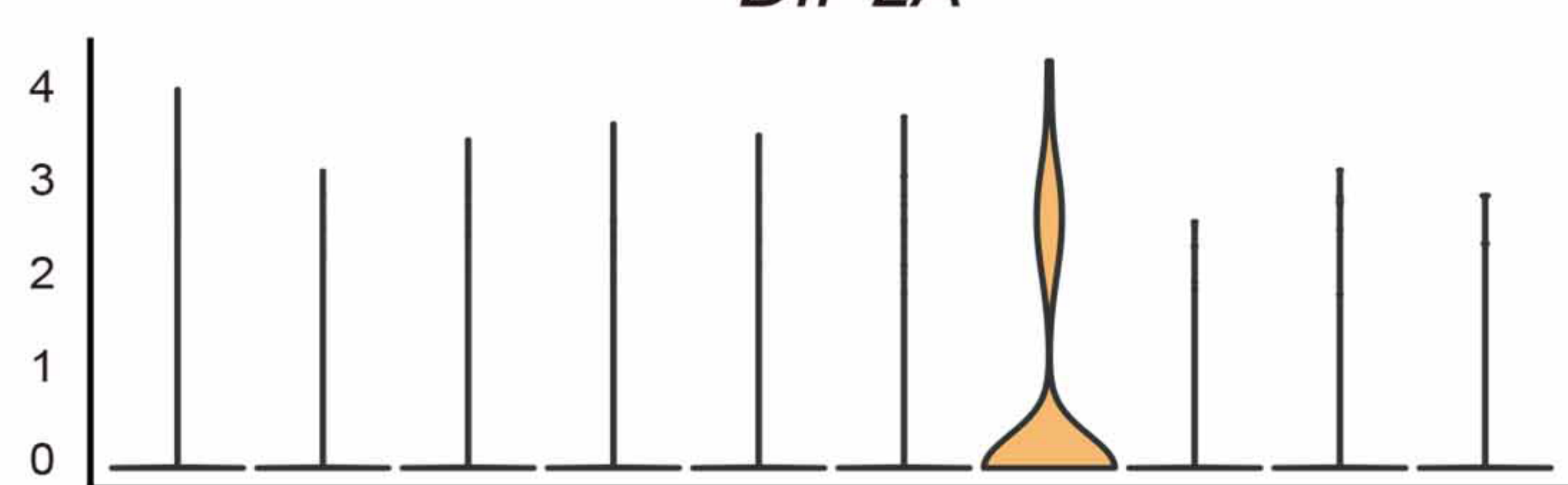

*PRMT2*

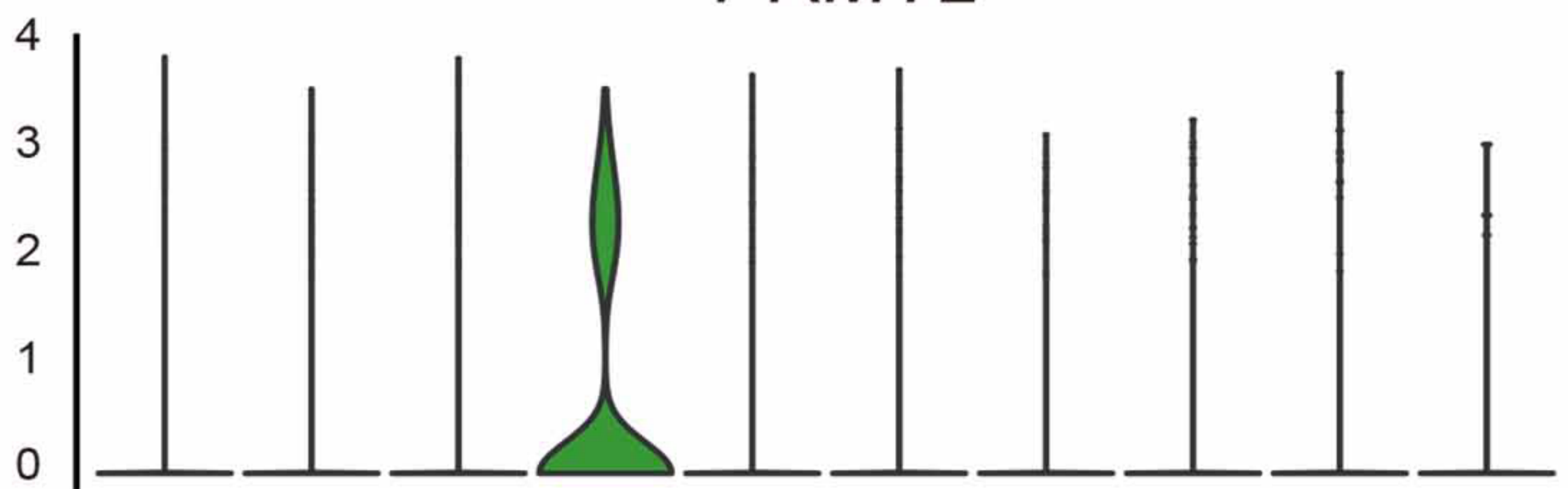

*BRWD1*

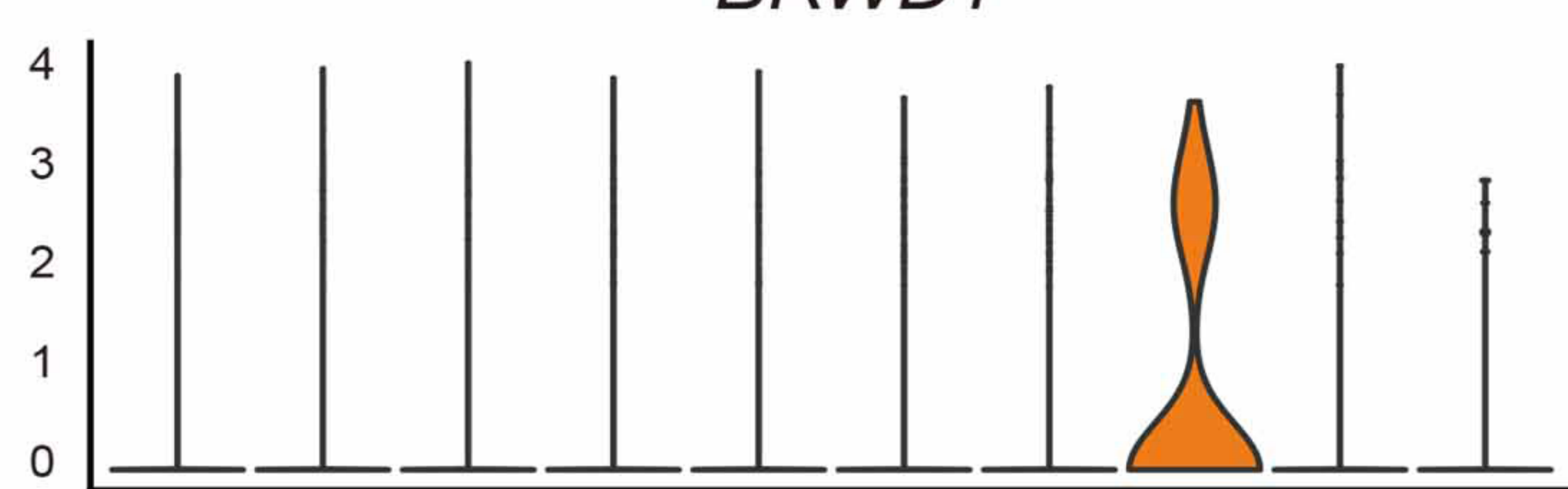

*USP25*

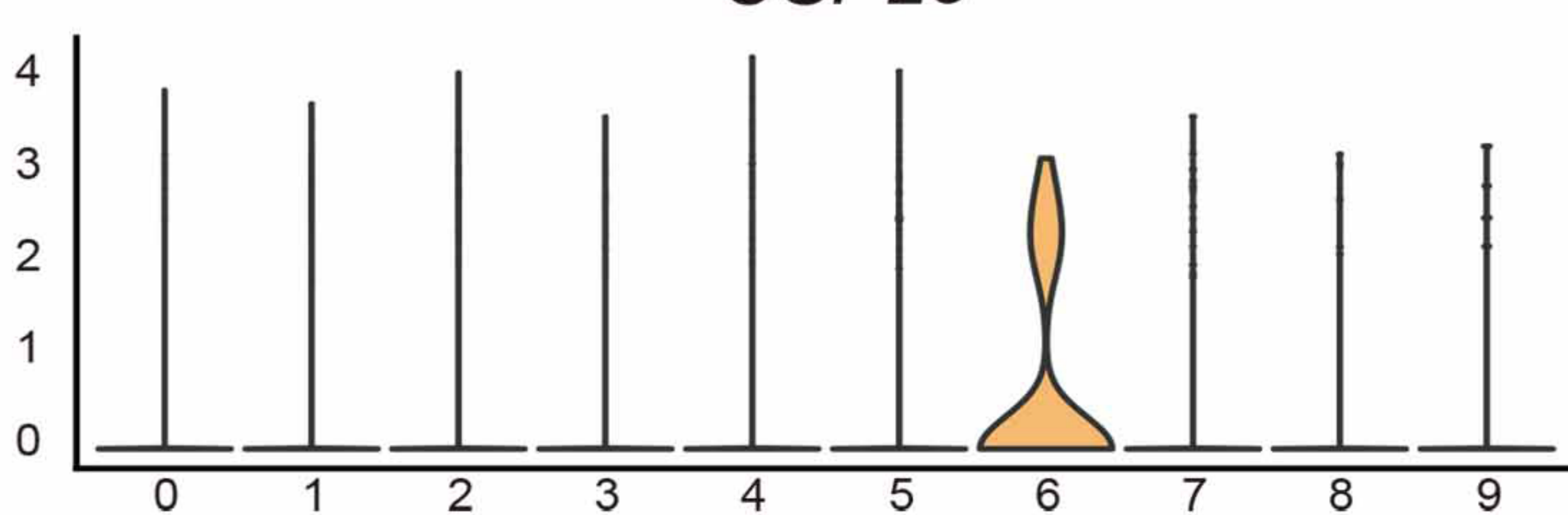

*PCP4*

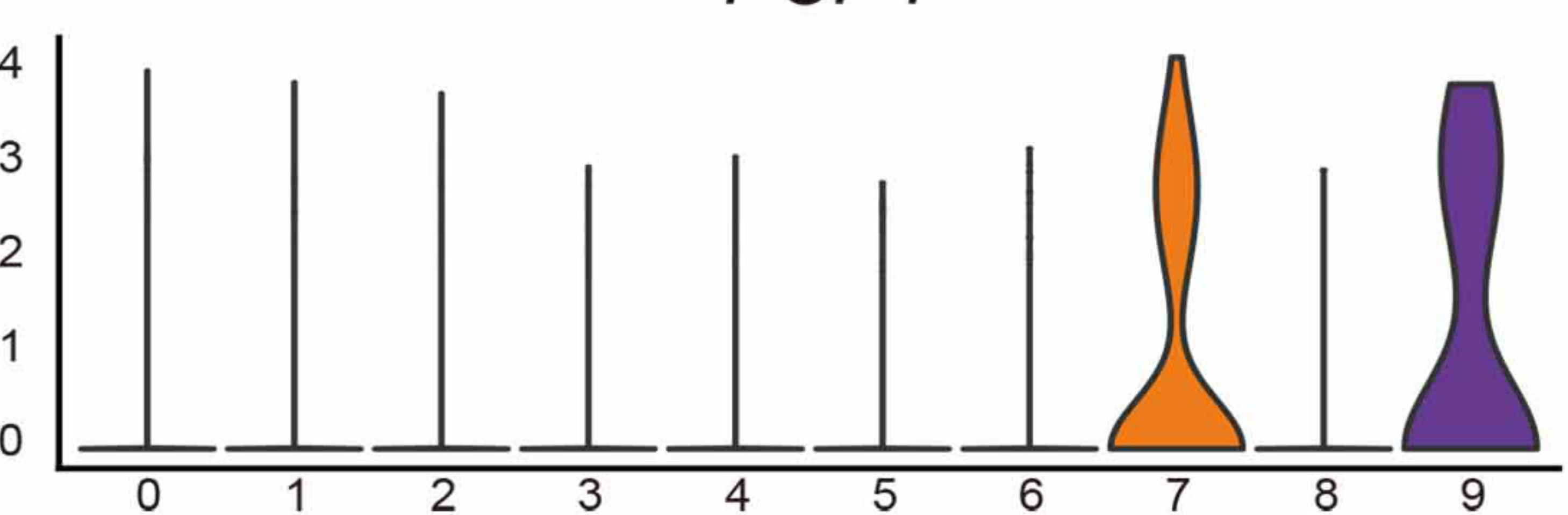

Supplement: Supplementary Figure 7 — Violin plots of genes on chromosome 21 specifically expressed in clusters. [file Image_7.pdf]
